# Self-adaptive nanozymes with enhanced multi-enzyme activities for sequential multimodal therapy of drug-resistant bacteria-infected wounds

**DOI:** 10.1038/s41467-026-73672-2

**Published:** 2026-05-28

**Authors:** Xiaoyong Zhang, Hang Yu, Kai Zhu, Yao Xiao, Yuxuan Gong, Dandan Che, Wanyi Chen, Guoxing You, Xiyun Yan, Quan Wang, Kelong Fan, Hong Zhou, Gan Chen

**Affiliations:** 1 Academy of Military Medical Sciences, Beijing, China; 2 CAS Engineering Laboratory for Nanozyme, Key Laboratory of Biomacromolecules (CAS), CAS Center for Excellence in Biomacromolecules, Institute of Biophysics, Chinese Academy of Sciences, Beijing, China

**Keywords:** Trauma, Nanobiotechnology, Biotechnology

## Abstract

Drug-resistant bacterium-infected wounds pose a serious clinical challenge, underscoring the need for therapeutic materials that respond to dynamic healing stages. Herein, we report a sequential multimodal platform embedding a self-adaptive IrPtCu nanozyme into a madecassoside-enriched hyaluronic acid hydrogel (HIPCM) for rapid bacterial eradication and accelerated wound healing. Leveraging trimetallic synergy and pH-adaptive reactive oxygen species (ROS) regulation, IrPtCu nanozyme exhibits strong oxidase, peroxidase, glutathione oxidase, and glutathione peroxidase-like activities, enabling efficient ROS generation and potent antibacterial performance. After disinfection, it switches to ROS scavenging through superoxide dismutase and catalase-like cascades, alleviating oxidative stress and cooperating with madecassoside to promote tissue repair. In a methicillin-resistant *Staphylococcus aureus* (*MRSA*)-infected mouse model, HIPCM demonstrates strong antibacterial efficacy, promotes M2 macrophage polarization and angiogenesis, and accelerates high-quality repair. Preclinical studies in Bama mini-pigs further confirm improved collagen deposition, hair follicle regeneration, and functional restoration. This work offers a comprehensive strategy integrating adaptive nanozymes and natural herbal medicines for treating drug-resistant wounds.

## Abstract (web summary)

Drug-resistant wound treatment requires adaptive response to the microenvironment. Here, authors develop a pH-responsive hydrogel therapy incorporating an IrPtCu nanozyme to sequentially eliminate pathogens and promote tissue regeneration.

## Introduction

Wound infection is a common and complex clinical problem that imposes substantial health burdens and medical challenges worldwide^1,2^. To date, broad-spectrum antibiotics combined with surgical debridement and flap transplantation represent the mainstay of clinical treatment^3^; unfortunately, conventional antibiotics are ineffective in managing complex infections caused by drug-resistant bacteria and also contribute to the development of bacterial resistance^4^. Furthermore, beyond bacterial eradication, the healing process of infected wounds involves dynamic stages of hemostasis, inflammation, proliferation, and remodeling, which are often overlooked in current treatments, ultimately impeding the functional recovery of native tissues and compromising patients’ quality of life^5^. Therefore, to address the clinical needs of healing wounds infected with drug-resistant bacteria, developing a comprehensive therapeutic strategy that meets the dynamic and complex requirements across different wound healing stages is crucial for promoting rapid and high-quality healing of infected wounds.

In the early stages of wound formation, primarily during hemostasis and inflammation, the progression of bacterial infection not only reduces the local microenvironmental pH and impairs the proliferation of local vascular endothelial cells but also further activates the body’s innate immune defense mechanism. This leads to the recruitment of inflammatory cells, the induction of an acute inflammatory response, and subsequent inhibition of wound healing^6–8^. Reactive oxygen species (ROS)-based therapy is a promising approach for wound disinfection^9^. However, excessive accumulation of ROS not only triggers a persistent inflammatory response, prolonging the inflammatory phase, but also hinders the proliferation and repair of tissue cells^10^. Thus, an adaptive therapeutic strategy that balances the dual requirements of ROS for both bactericidal activity and wound healing is imperative^8,11^. Additionally, due to the structural and compositional complexity of skin tissue, the regeneration of intact skin encompassing not only collagen deposition and angiogenesis but also the restoration of skin appendages such as hair follicles remains a major unmet challenge. Therefore, the design of an infected wound microenvironment-adaptive therapy capable of simultaneously eliminating bacteria, restoring redox and immune homeostasis, and promoting tissue regeneration is essential for achieving rapid functional wound healing.

Nanozymes are widely applied in biomedical research owing to their facile preparation, robust physicochemical stability, and multi-enzyme activities^12–14^, including peroxidase-like (POD-like), oxidase-like (OXD-like), glutathione peroxidase-like (GSH-Px-like), catalase-like (CAT-like), and superoxide dismutase-like (SOD-like) activities. These properties enable nanozymes to simultaneously generate or scavenge ROS, thereby maintaining intracellular ROS homeostasis. Currently, nanozymes are commonly used to construct a series of antibacterial systems, which are more conducive to preventing the emergence of bacterial resistance^15,16^. Based on their compositional and structural characteristics, commonly used antibacterial nanozymes can be classified into several main categories, including noble metal nanozymes (Au, Ag, Pt, Cu), metal oxide nanozymes (ZnO, Fe_2_O_3_), transition metal dichalcogenide/carbide nanozymes (MoS_2_, MXene), and hybrid/composite nanozymes^17–19^. However, their low catalytic activity, lack of microenvironment adaptability, and unimodal antibacterial mechanism are the key factors limiting their antibacterial efficiency and therapeutic applications^20^. Despite extensive efforts in recent years to enhance the catalytic performance of Pt-based nanozymes (with the Pt-Cu system recognized as one of the most promising configurations), their ROS generation efficiency still requires further improvement^21–24^. Regulating the electronic structure and optimizing catalytic pathways through the synergistic effects of multiple metals represents a highly promising strategy for boosting enzymatic activity^25–27^. Accordingly, multimetallic nanozymes synthesized based on the Pt-Cu framework are expected to achieve synergistically enhanced multi-enzyme activities and potent antibacterial performance.

On the other hand, madecassoside (MA) is a pentacyclic triterpenoid saponin extracted from *Centella asiatica*, with diverse pharmacological activities^28^, including wound healing promotion, antitumor effects, and immunomodulatory properties. Furthermore, MA has been shown to accelerate burn wound healing by promoting collagen deposition, enhancing angiogenesis and re-epithelialization, and facilitating hair follicle development^29–31^, rendering it widely used in wound healing and scar management. Notably, while the efficacy of MA in common wound repair is well documented, its synergistic application with functional nanozymes for addressing the refractory microenvironment of drug-resistant bacterium-infected wounds has rarely been reported.

**Fig. 1:**
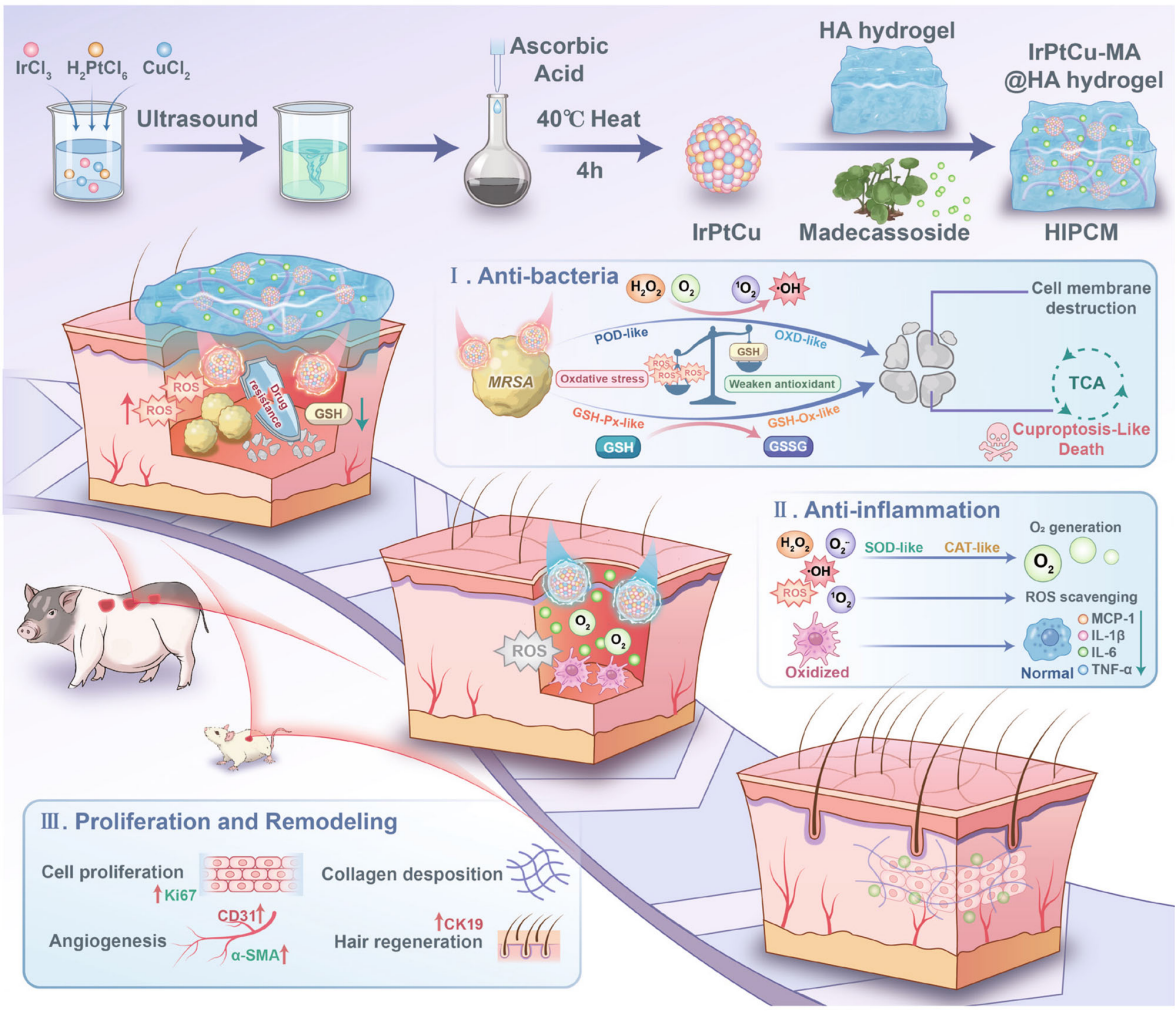
Schematic illustration of the design and application of HIPCM for the treatment of *MRSA*-infected wounds and full-thickness skin defects.

Herein, we report the development of a multifunctional therapeutic hydrogel system (HIPCM) composed of an IrPtCu nanozyme and MA, which delivers adaptive, sequential antibacterial, anti-inflammatory, and pro-regenerative interventions that align with wound pathophysiology (Fig. 1). Initially, under acidic, infection-associated conditions, HIPCM efficiently catalyzes the generation of excessive ROS while depleting glutathione (GSH), thereby damaging bacterial membranes and disrupting the tricarboxylic acid (TCA) cycle to exert robust antibacterial activity. As the wound microenvironment transitions toward neutrality, HIPCM switches its function to scavenge excess ROS and reactive nitrogen species (RNS), exerting antioxidant effects and alleviating local inflammation. Finally, in regeneration-favorable microenvironments, HIPCM promotes collagen deposition, cell proliferation, and angiogenesis, significantly accelerating the healing of *MRSA*-infected wounds. Notably, to meet the requirements of clinical translation, evaluations were performed using a full-thickness skin defect model in Bama mini-pigs. HIPCM retained its therapeutic efficacy and achieved high-quality wound closure, confirming its robust reparative performance and favorable biocompatibility. Overall, HIPCM provides a clinically translatable strategy for comprehensive wound management (including but not limited to infected wounds) by coordinating antibacterial action, immunomodulation, and tissue regeneration in a stage-specific manner.

## Results

### Preparation and characterization of HIPCM

**Fig. 2:**
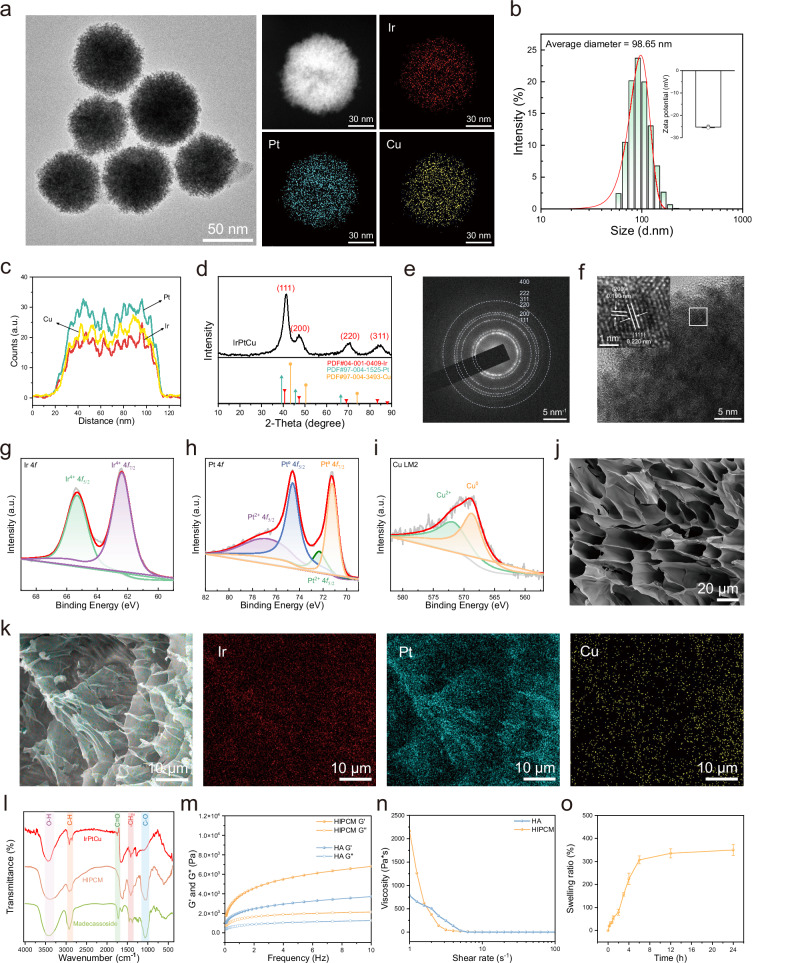
Characterization of IrPtCu nanozyme and HIPCM. **a** HRTEM image (Scale bars, 50 nm) of IrPtCu nanozyme coupled with corresponding EDS elemental mapping images (Scale bars, 30 nm) revealing the spatial distributions of Ir, Pt, and Cu (*n* = 3 independent replicates). **b** Hydrodynamic size distribution and zeta potential of IrPtCu nanozyme. **c** Elemental line-scanning profile across an individual IrPtCu nanozyme. **d** XRD pattern of the crystalline IrPtCu nanozyme phase. **e** SAED pattern of IrPtCu nanozyme (Scale bars, 5 nm^−1^). **f** HRTEM image of IrPtCu nanozyme (*n* = 3 independent replicates; Scale bars, 5 nm and 1 nm). **g-i** XPS spectra of IrPtCu nanozyme: **g** Ir 4 f, **h** Pt 4 f, and **i** Cu 2p core levels. **j** SEM image (*n* = 3 independent replicates; Scale bars, 20 μm) of HIPCM and **k** corresponding EDS elemental mapping images (Scale bars, 10 μm) for Ir, Pt, and Cu. **l** FTIR spectra of IrPtCu nanozyme, madecassoside, and HIPCM. **m** Frequency sweep tests of the different hydrogels. **n** Viscosity of the different hydrogels. **o** Swelling curves of the HIPCM. Source data are provided as a Source Data file.

The preparation of IrPtCu nanozyme, using IrCl_3_, H_2_PtCl_6_, and CuCl_2_ as metal precursors and ascorbic acid as the reducing agent, is schematically illustrated in Fig. 1. The Ir content was optimized by adding IrCl_3_ at various concentrations during the synthesis, following the previously established PtCu formulation^32^, and the POD- and CAT-like activities of the resulting products were subsequently evaluated. The IrPtCu nanozyme synthesized with a final IrCl_3_ concentration of 10 mM showed the best overall enzyme-mimicking performance and was therefore selected for subsequent studies (Supplementary Fig. 1). For comparison, the PtCu nanozyme was prepared in parallel using H_2_PtCl_6_ and CuCl_2_ as precursors, with ascorbic acid as the reducing agent. High-resolution transmission electron microscopy (HRTEM) characterization revealed that the IrPtCu nanozyme exhibited a well-defined mulberry-like morphology (Fig. 2a). Dynamic light scattering (DLS) and zeta potential measurements indicated that IrPtCu nanozyme had good aqueous dispersibility (zeta potential: − 25.25 mV) with an average hydrodynamic diameter of ~98.65 nm (Fig. 2b). Energy-dispersive X-ray spectroscopy (EDS) elemental mapping (Fig. 2a) showed that Ir, Pt, and Cu were uniformly distributed throughout the structure, confirming the successful formation of the trimetallic IrPtCu nanozyme. Line-scan analysis revealed a heterostructure within the hybrid system, and the distribution of elemental Ir coincided with that of Pt, consistent with the more favorable formation of the IrPt hybrid relative to the PtCu hybrid (Fig. 2c). On the basis of inductively coupled plasma-optical emission spectrometry (ICP-OES) analysis (Supplementary Table 1), the apparent atomic ratio of Ir:Pt:Cu in IrPtCu nanozyme was determined to be ~1.21:1.00:3.27. Furthermore, the crystal structure of the IrPtCu nanozyme was characterized by X-ray powder diffraction (XRD). The typical diffraction peaks indicated the formation of an intermetallic phase, with no impurity peaks observed (Fig. 2d). The selected area electron diffraction (SAED) pattern further confirmed that the interplanar spacings were consistent with those of the (111), (200), (220), and (311) planes of the cubic structure (Fig. 2e). The HRTEM image clearly showed lattice fringes with interplanar spacings of 0.220 and 0.190 nm (Fig. 2f), which matched well with the (200) and (111) planes of the IrPtCu nanozyme crystal. The valence states of the resulting IrPtCu nanozyme were determined via X-ray photoelectron spectroscopy (XPS). Ir^4+^ was confirmed by the peaks at 62.37 eV and 65.30 eV, which were consistent with the binding energy signature of standard IrO_2_ (Fig. 2g). The peaks located at 71.23 eV and 74.58 eV corresponded to metallic Pt^0^, and additional peaks at 72.33 eV (4*f*_7/2_) and 76.73 eV (4*f*_5/2_) corresponded to surface Pt^2+^ (Fig. 2h). In the Cu 2*p* XPS spectrum, the peak at 571.59 eV (2*p*_3/2_) was assigned to Cu^2+^, and a peak at 568.57 eV (2*p*_3/2_) was assigned to Cu^0^ (Fig. 2i). These multivalent metal species show great potential as active sites for ROS production. In summary, we successfully prepared an IrPtCu nanozyme with a well-defined intermetallic structure and uniform morphology.

To establish a sequential multimodal therapeutic platform, the IrPtCu nanozyme was encapsulated within a hyaluronic acid (HA) hydrogel enriched with MA. The resulting composite system was designated HIPCM. Scanning electron microscopy (SEM) images of the lyophilized HIPCM hydrogel revealed a uniformly interconnected porous structure in its cross-section (Fig. 2j). EDS mapping confirmed the homogeneous distribution of Ir, Pt, and Cu within the hydrogel matrix, indicating successful and uniform loading of the IrPtCu nanozyme (Fig. 2k). On the basis of these structural and compositional findings, Fourier transform infrared (FTIR) spectroscopy was used to investigate the chemical structure of HIPCM (Fig. 2l). The significant increase in the relative intensity and redshift of the O − H absorption band from 3466 cm^−1^ to 3395 cm^−1^ is associated with the introduction of O − H groups from both the MA and the IrPtCu nanozyme surfaces, suggesting crosslinking via hydrogen-bonding interactions among the components. The C = O absorption peak at 1748 cm^−1^ is assignable to the ester carbonyl stretching vibration of MA, confirming its successful incorporation into the HA network. The enhanced peak intensity at 1415 cm^−1^ is attributable to the introduction of organic ligands on the IrPtCu nanozyme surface. Collectively, these spectral changes confirm the successful loading of both MA and IrPtCu nanozyme within the HA hydrogel, forming a stable composite system primarily stabilized by hydrogen bonding.

To evaluate whether the introduction of the IrPtCu nanozyme affected the gelation of the HA hydrogel, a rheometer was used to perform dynamic rheological analysis. Time-sweep dynamic rheology tests demonstrated that the storage modulus (G’) was significantly greater than the loss modulus (G”) (Fig. 2m), confirming the formation of a stable, cross-linked three-dimensional network. This elastic-dominated behavior endows the HIPCM with high shape adaptability, facilitating conformal coverage of irregular wounds. Shear-rate sweep tests revealed pronounced shear-thinning behavior: the viscosity decreased by two orders of magnitude as the shear rate increased from 0.1 s^−1^ to 100 s^−1^ (Fig. 2n). This property ensures both injectability and rapid self-recovery at the wound site. Next, the swelling profile of the HIPCM were evaluated under physiological conditions. HIPCM reached equilibrium water absorption within 24 h (Fig. 2o), a characteristic beneficial for absorbing wound exudate and maintaining a moist healing environment. In addition, the hemolysis rate of hydrogels was evaluated, and the results consistently revealed negligible hemolytic activity in HIPCM (Supplementary Fig. 2). Furthermore, CCK-8 assays performed on human umbilical vein endothelial cells (HUVECs) exposed to HIPCM extracts revealed that cell viability exceeded 90% at 24, 36, and 72 h (Supplementary Fig. 3), demonstrating favorable cytocompatibility.

### Multi-enzyme activities of IrPtCu nanozyme

**Fig. 3:**
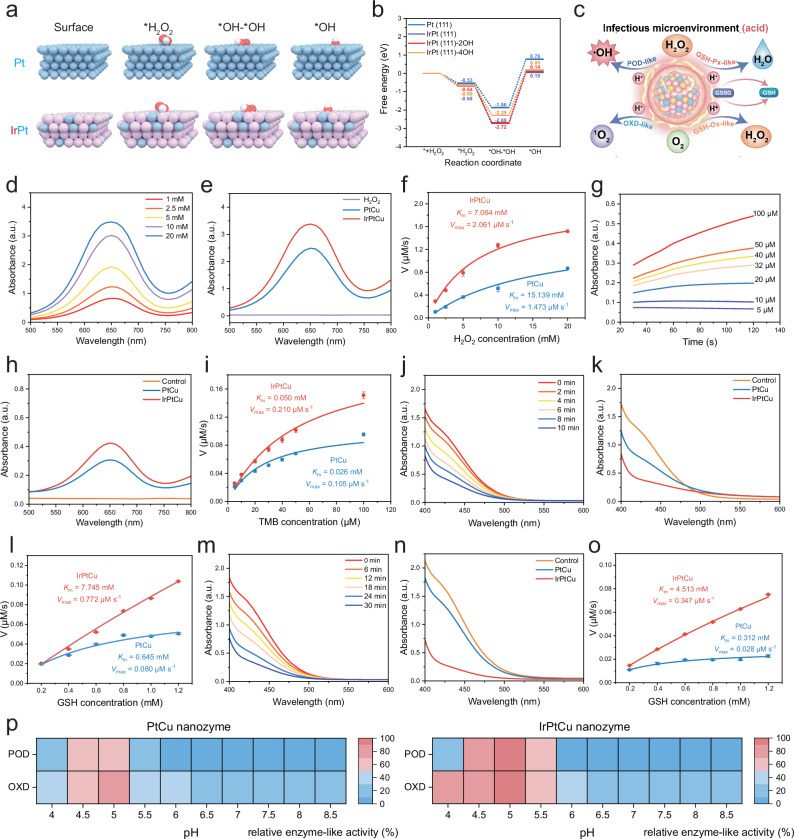
ROS production and GSH depletion activities of the nanozymes. **a** Surface models and **b** free energy diagrams of Pt as the active site in PtCu models and Ir and Pt as the active sites in IrPtCu nanozyme models during the POD-like reaction process. **c** Antibacterial mechanisms of IrPtCu nanozyme in an infectious microenvironment. **d** Time-dependent UV/Vis absorption changes of H_2_O_2_ and TMB solutions upon addition of IrPtCu nanozyme. **e** UV/Vis absorption of reaction solutions containing TMB + H_2_O_2_, PtCu + TMB + H_2_O_2_, and IrPtCu nanozyme + TMB + H_2_O_2_ after a 1-min reaction. **f** Michaelis‒Menten kinetic analysis of PtCu and IrPtCu nanozymes for POD-like activity with H_2_O_2_ as the substrate (*n* = 3 independent replicates, data are presented as mean values ± SD). **g** Time-dependent UV/Vis absorption changes of the TMB solution upon addition of the IrPtCu nanozyme. **h** UV/Vis absorption of reaction solutions containing TMB alone, PtCu + TMB, and IrPtCu nanozyme + TMB after a 1-min reaction. **i** Michaelis‒Menten kinetic analysis of PtCu and IrPtCu nanozymes for OXD-like activity with TMB as the substrate (*n* = 3 independent replicates, data are presented as mean values ± SD). **j** Time-dependent UV/Vis absorption changes of H_2_O_2_ and GSH solutions upon addition of the IrPtCu nanozyme. **k** UV/vis absorption of solutions containing DTNB + H_2_O_2_, PtCu+ H_2_O_2_ + DTNB, and IrPtCu nanozyme + H_2_O_2_ + DTNB after a 10-min reaction. **l** Michaelis‒Menten kinetic analysis of the GSH-Px-like activity of PtCu and IrPtCu nanozymes with H_2_O_2_ and GSH as the substrates (*n* = 3 independent replicates, data are presented as mean values ± SD). **m** Time-dependent UV/Vis absorption changes of the GSH solution upon addition of IrPtCu nanozyme. **n** UV/Vis absorption of solutions containing DTNB, PtCu + DTNB, and IrPtCu nanozyme + DTNB after a 30-min reaction. **o** Michaelis‒Menten kinetic analysis of the GSH-Ox-like activity of PtCu and IrPtCu nanozymes with GSH as the substrate (*n* = 3 independent replicates, data are presented as mean values ± SD). **p** Effect of pH on the enzyme-like activities of PtCu and IrPtCu nanozymes. Source data are provided as a Source Data file.

To elucidate the influence of Ir incorporation on the catalytic activities of the PtCu system, DFT calculations were performed using the Vienna ab initio simulation package to clarify the atomic-level POD-like mechanism of the IrPtCu nanozyme, as illustrated in Fig. 3a. Two computational surface models representing Pt (111) and IrPt (111) were constructed to clarify their microscopic electron structures and enzyme‑like catalytic mechanisms. Hydroxylated surface states (2OH and 4OH coverages) were included to simulate aqueous reaction conditions. The catalytic reaction fundamentally involves the adsorption and desorption of intermediates, and appropriate interactions between metal sites and substrates or intermediates are critical for high catalytic activity^33^. These models enabled mapping of the full reaction sequence, including H_2_O_2_ adsorption, activation, O − O bond cleavage, and ·OH release, via calculated free-energy changes (ΔG) to identify the rate-limiting steps. As summarized in Fig. 3b, Pt (111) facilitates H_2_O_2_ adsorption (ΔG = −0.531 eV) and dissociation (ΔG = −1.332 eV), consistent with its known POD-like activity, but ·OH release (*OH − *OH → *OH + ·OH) is energetically demanding (ΔG = 2.644 eV). The incorporation of Ir into Pt (111) markedly improved the energetics: IrPt (111) exhibited stronger H_2_O_2_ binding (ΔG = −0.684 eV) and easier O − O bond scission (ΔG = −2.014 eV), leading to more efficient ·OH formation. Under high hydroxyl coverage (IrPt (111) − 4OH), ·OH release is further facilitated, lowering the ΔG barrier to 2.488 eV, a reduction of 0.156 eV compared with Pt (111). Based on the DFT results, a synergistic interaction between the Ir and Pt sites is anticipated. Ir modulates the d-band center and enhances oxygen‑binding affinity, accelerating H_2_O_2_ adsorption and dissociation. As the reaction proceeds, the Ir-Pt hybrid bimetallic surface becomes increasingly prone to oxide-phase formation. This, in turn, triggers a cooperative interplay between lateral adsorbate repulsion and hydrogen-bond network reorganization, thereby attenuating the ·OH–metal interaction and facilitating partial ·OH desorption. These ·OH species exhibit exceptionally high oxidative activity, endowing the IrPtCu nanozyme with substantial potential for antibacterial applications.

To validate the catalytic properties of IrPtCu and PtCu nanozymes predicted by DFT calculations, we evaluated their capacities for ROS generation (POD-like and OXD-like activities) and for glutathione depletion (GSH-Px-like and GSH-Ox-like activities) in a weakly acidic (pH 5.5), infection-mimicking microenvironment (Fig. 3c). First, for ROS generation, POD-like activity can catalyze the decomposition of H_2_O_2_ into ·OH, whereas OXD-like activity can catalyze the conversion of O_2_ into ·O_2_^−^. Both POD-like activity and ·OH generated by OXD-like activity can catalyze the oxidation of 3,3’,5,5’-tetramethylbenzidine (TMB) to blue oxidized TMB (oxTMB). As shown in Fig. 3p, the IrPtCu nanozyme exhibited almost no discernible POD-like activity at near‑neutral pH, whereas the catalytic activity markedly increased as the pH decreased. These results indicate that the nanozymes are responsive to acidic microenvironments (pH 4.5-5.5). Upon addition of the IrPtCu nanozyme, the characteristic oxTMB absorption at 652 nm increased over time (Fig. 3d). As shown in Fig. 3e, IrPtCu nanozyme exhibited stronger POD-like activity than the PtCu nanozyme. The TMB colorimetric reaction for these nanozymes exhibited typical Michaelis‒Menten kinetics with H_2_O_2_ as the reaction substrate at pH 5.5. The Michaelis‒Menton constant (*K*_m_) value and maximum reaction rate (*V*_max_) of IrPtCu nanozyme were 7.064 mM and 2.061 × 10^−6 ^M s^−1^, respectively (Fig. 3f). Under the same conditions, the *K*_m_ value and *V*_max_ of PtCu nanozyme were 15.139 mM and 1.473 × 10^−6 ^M s^−1^, respectively. The lower *K*_m_ indicates that IrPtCu nanozyme has a higher H_2_O_2_ affinity than the PtCu nanozyme, whereas the higher *V*_max_ indicates that the IrPtCu nanozyme has a faster maximum catalytic rate than the PtCu nanozyme. The catalytic efficiency of the IrPtCu nanozyme was further compared with other state-of-the-art nanozymes, revealing its exceptional substrate affinity and elevated intrinsic catalytic activity (Supplementary Fig. 4). Analogous to its POD‑like behavior, the IrPtCu nanozyme exhibited robust OXD‑like activity only under acidic conditions (Fig. 3p). Upon addition of the IrPtCu nanozyme to the TMB solution, the characteristic absorption increased over time (Fig. 3g). As shown in Fig. 3h, the IrPtCu nanozyme exhibited stronger OXD‑like activity than the PtCu nanozyme. With TMB as the reaction substrate, the *K*_m_ value and *V*_max_ of the IrPtCu nanozyme were 0.050 mM and 0.210 × 10^−6 ^M s^−1^, respectively, whereas those of the PtCu nanozyme were 0.026 mM and 0.105 × 10^−6 ^M s^−1^, respectively (Fig. 3i). The higher *V*_max_ indicates that the IrPtCu nanozyme has greater OXD-like catalytic activity than the PtCu nanozyme. These results indicate that, owing to the exceptional substrate affinity and catalytic activity of the Ir-Pt hybrid bimetallic surface, the IrPtCu nanozyme efficiently catalyzes ROS generation, consistent with the DFT results shown in Fig. 3b.

A negative correlation exists between the therapeutic efficacy of ROS-based therapies and the level of reduced GSH in the bacterial microenvironment^34,35^. Given the pivotal role of GSH in sustaining bacterial growth and orchestrating antioxidant defense mechanisms, we systematically evaluated the dual enzyme-mimicking activities of the IrPtCu nanozyme in catalyzing GSH oxidation, including both GSH-Px- and GSH-Ox-like activities. Using 5,5ʼ-dithiobis (2-nitrobenzoic acid) (DTNB) as a probe, we monitored GSH depletion via the 410 nm UV absorption peak of the reaction product 2-nitro-5-thiobenzoate (TNB). The GSH-Px-like activity was characterized by the removal of dissolved oxygen under a nitrogen atmosphere and the addition of H_2_O_2_ (Fig. 3k), whereas the GSH-Ox-like activity was assessed without H_2_O_2_ (Fig. 3n). Compared with the PtCu nanozyme, the IrPtCu nanozyme significantly reduced the characteristic absorbance of the GSH-DTNB adduct. In the presence of H_2_O_2_, GSH was almost completely consumed within 10 min (Fig. 3j), indicating the strong GSH‑Px‑like activity. Similarly, in the absence of H_2_O_2_, GSH was depleted within 30 min of incubation (Fig. 3m), demonstrating robust GSH-Ox-like activity. Furthermore, the *V*_max_ values of the IrPtCu nanozyme for GSH-Px-like (0.772 × 10^−6 ^M s^−1^) and GSH-Ox-like (0.347 × 10^−6 ^M s^−1^) activities were notably greater than those of the PtCu nanozyme under comparable conditions (Fig. 3l, o). These findings indicate that the IrPtCu nanozyme generates ROS while efficiently depleting GSH, thereby holding promise for enhancing ROS‑mediated antibacterial activity.

In summary, the IrPtCu nanozyme emerges as a Janus nanozyme with POD-, OXD-, GSH-Px-, and GSH-Ox-like properties under acidic conditions, continuously generating ROS while depleting GSH. This dual functionality positions the IrPtCu nanozyme as a promising candidate for antimicrobial applications against drug-resistant bacterial infections.

**Fig. 4:**
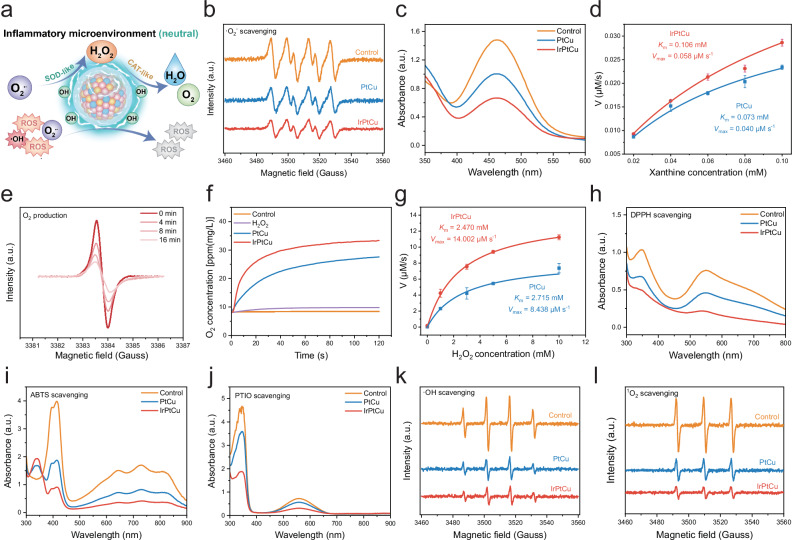
ROS scavenging activities of the nanozymes. **a** ROS scavenging ability of IrPtCu nanozyme. **b** SOD-like activities of PtCu and IrPtCu nanozymes in reducing superoxide, as demonstrated by ESR spectra. **c** UV/Vis absorption of reaction solutions containing WST-8 alone, PtCu + WST-8, and IrPtCu nanozyme + WST-8 after a 20-min reaction. **d** Michaelis‒Menten kinetic analysis of the SOD-like activity of PtCu and IrPtCu nanozymes (*n* = 3 independent replicates, data are presented as mean values ± SD). **e** ESR spectra of PDT over time before and after addition of IrPtCu nanozyme. **f** O_2_ generation. **g** Michaelis‒Menten kinetic analysis of the CAT-like activity of PtCu and IrPtCu nanozymes (*n* = 3 independent replicates, data are presented as mean values ± SD). **h** DPPH, **i** ABTS, and **j** PTIO scavenging activities of PtCu and IrPtCu nanozymes. **k** ·OH and **l** ^1^O_2_ scavenging by PtCu and IrPtCu nanozymes, as detected by ESR spectroscopy. Source data are provided as a Source Data file.

After confirming the pH‑responsive ROS‑generation and GSH depletion activities of the IrPtCu nanozyme in infection‑mimicking acidic environments, we next examined its ROS‑scavenging activity in inflammatory tissues (neutral conditions). We hypothesized that the IrPtCu nanozyme can alleviate hypoxia and intracellular oxidative stress to promote cell proliferation in an inflammatory environment (pH 7.4) by mimicking SOD-like activity to convert ·O_2_⁻ to H_2_O_2_ and decomposing excess H_2_O_2_ into H_2_O and O_2_ via CAT-like activity (Fig. 4a). SOD is a crucial enzyme that protects cellular components from oxidative damage by degrading ·O_2_⁻ and maintaining ROS homeostasis^36^. Using a classical KO_2_ system to generate ·O_2_⁻ in an aprotic solvent with a crown ether and employing BMPO (5-tert-butoxycarbonyl-5-methyl-1-pyrroline-N-oxide) as a spin-trapping agent, we observed significant reductions in electron spin resonance (ESR) signal intensities upon addition of the IrPtCu or PtCu nanozyme (Fig. 4b). WST-8 reacts specifically with ·O_2_⁻ to generate a water‑soluble formazan dye, whose formation can be quantitatively monitored via spectrophotometric analysis. The rate of formazan production provides a reliable measure of SOD-like activity. As shown in Fig. 4c, the IrPtCu nanozyme exhibited superior catalytic efficiency in scavenging ·O_2_⁻ compared with the PtCu nanozyme, with *K*_m_ and *V*_max_ values of 0.106 mM and 0.058 × 10^−6 ^M s^−1^, respectively, whereas those of the PtCu nanozyme were 0.073 mM and 0.040 × 10^−6 ^M s^−1^, respectively (Fig. 4d). Similarly, CAT-like activity was assessed by measuring O_2_ generation from H_2_O_2_ decomposition (Fig. 4e, f). As indicated in Fig. 4g, the IrPtCu nanozyme efficiently catalyzed the breakdown of H_2_O_2_ into O_2_, with *K*_m_ and *V*_max_ values of 2.470 mM and 14.002 × 10^−6 ^M s^−1^, respectively. Furthermore, the IrPtCu nanozyme exhibited significantly greater scavenging activity toward both nitrogen-centered radicals (DPPH and ABTS) and oxygen-centered radicals (PTIO) than the PtCu nanozyme under neutral conditions (Fig. 4h-j). Consistently, ESR-based measurements of ·OH and ^1^O_2_ also indicated superior ROS scavenging efficiency for the IrPtCu nanozyme compared with the PtCu nanozyme (Fig. 4k, l). Collectively, these findings highlight the ability of the IrPtCu nanozyme to effectively neutralize ROS and RNS in neutral tissue environments, thereby maintaining redox homeostasis. This property is expected to foster a microenvironment conducive to cell proliferation and angiogenesis during wound treatment, underscoring its promise for wound healing applications.

In conclusion, the IrPtCu nanozyme exhibits multi-enzyme, pH‑adaptive catalytic activities tailored to distinct biological contexts. Its ability to generate ROS under acidic conditions while efficiently scavenging ROS under neutral conditions highlights its unique bidirectional reactivity and underscores its potential for next-generation biomedical interventions, ranging from potent antibacterial strategies to precise modulation of oxidative stress.

### In vitro antibacterial activitiy

**Fig. 5:**
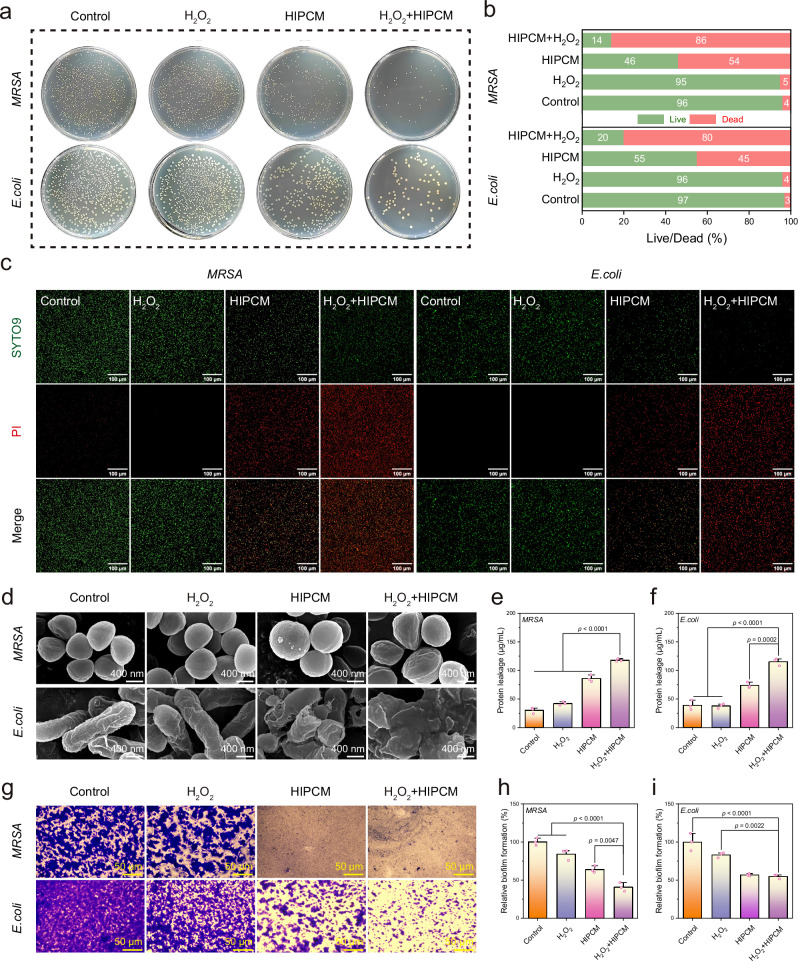
In vitro antibacterial activitiy of HIPCM. **a** Representative photographs of bacterial colonies. **b** Quantitative analysis of dead (red) and live (green) *MRSA* and *E. coli* (1: Control, 2: H_2_O_2_, 3: HIPCM, 4: H_2_O_2_ + HIPCM) (*n* = 3 independent replicates) and **c** fluorescence images (Scale bars, 100 μm). **d** Typical SEM images of *MRSA* and *E. coli* after various treatments (Scale bars, 400 nm). Protein leakage of **e** *MRSA* and **f** *E. coli* after different treatments (*n* = 3 independent replicates). **g** Representative photographs of inhibited biofilms after different treatments stained with crystal violet (Scale bars, 50 μm). **h** Inhibition rates of *MRSA*-formed and **i** *E. coli*-formed biofilms (*n* = 3 independent replicates). All data are expressed as the mean values ± SD. Statistical significance was determined using one-way ANOVA for multiple-group comparisons, followed by Tukey’s two-tailed post-hoc test for pairwise analysis, all tests were two-sided. Source data are provided as a Source Data file.

The IrPtCu nanozyme component of the HIPCM system can generate ROS and deplete GSH in the mildly acidic microenvironment associated with bacterial infections, thereby enabling highly efficient bactericidal activity. We therefore performed comprehensive in vitro experiments to evaluate the antibacterial activity of HIPCM against *MRSA* and *E. coli*. First, we compared the antibacterial activities of PBS (control), H_2_O_2_, HIPCM, and HIPCM + H_2_O_2_ against *MRSA* and *E. coli*. As shown in Fig. 5a, the total number of colonies in the HIPCM and HIPCM + H_2_O_2_ groups was significantly lower than that in the control and H_2_O_2_ groups (Supplementary Fig. 5, 6), which is attributable to the presence of the IrPtCu nanozyme. live-dead staining further confirmed these findings: the HIPCM + H_2_O_2_ group exhibited the highest percentage of dead bacteria, with almost no live bacteria observed (Fig. 5b, c). Notably, both bacterial culture plates and bacterial survival rates showed that the pure H_2_O_2_ (100 μM) group had negligible bactericidal activity, whereas the HIPCM + H_2_O_2_ group had almost no colonies, confirming the high antibacterial activity of the IrPtCu nanozyme against *MRSA* and *E. coli*. Additionally, SEM was employed to observe bacterial morphological changes. As shown in Fig. 5d, compared with the smooth, intact cell walls of untreated *MRSA* and *E*. *coli*, bacteria in the HIPCM + H_2_O_2_ group exhibited morphological deformation with rough, sunken membranes. Furthermore, we assessed the degree of protein leakage from *MRSA* and *E. coli* following treatment. HIPCM treatment increased protein leakage, and the HIPCM + H_2_O_2_ group showed a substantial increase (Fig. 5e, f), demonstrating that HIPCM exerts strong bactericidal effects by disrupting membrane integrity. Bacterial biofilms, comprising structured communities embedded in an extracellular polymeric substance matrix of polysaccharides, proteins, and DNA, create a protective niche for bacteria that markedly hinders wound repair^37,38^. We therefore evaluated the antibiofilm activity of HIPCM via crystal violet staining. As shown in Fig. 5g, HIPCM treatment substantially reduced crystal violet retention, with the HIPCM + H_2_O_2_ combination inducing the most pronounced biofilm loss (Fig. 5h, i). Furthermore, ROS production was detected by staining bacteria with the 2’,7’-dichlorodihydrofluorescein (DCFH-DA) probe. Supplementary Fig. 7 shows that both the HIPCM and HIPCM + H_2_O_2_ groups produced green fluorescence, and the HIPCM + H_2_O_2_ group exhibited markedly stronger green fluorescence than the HIPCM group, indicating enhanced ROS signal intensity under oxidative conditions.

In conclusion, HIPCM exhibits robust antibacterial and antibiofilm activities under mildly acidic conditions, highlighting its high potential for promoting the healing of drug-resistant bacterium-infected wounds.

### HIPCM exhibits antibacterial activity via ROS generation mediated by multi-enzyme activities and a cuproptosis-like mechanism

**Fig. 6:**
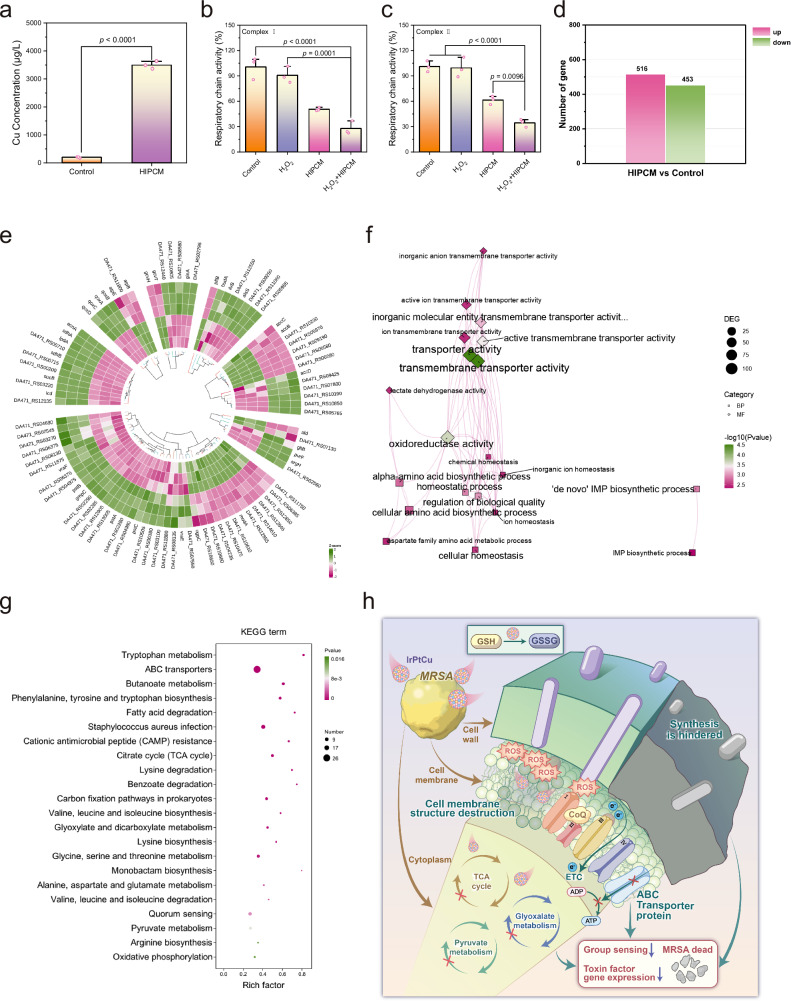
Antibacterial mechanisms of HIPCM. **a** Intracellular copper content in *MRSA* (*n* = 3 independent replicates). Activities of respiratory chain complex I **b** and complex II **c** in *MRSA* (*n* = 3 independent replicates). **d** Number of DEGs. **e** Heatmap of DEGs involved in the bacterial metabolism pathways and a summary of genes related to cuproptosis-like death, oxidative stress, and cell wall-specific disruption. **f** GO enrichment analysis of DEG functions. **g** KEGG enrichment analysis scatter plot. **h** Schematic diagram of the antibacterial mechanisms. All data are expressed as the mean values ± SD. Statistical significance was determined using the Student’s t-test for two-group comparisons, and (b, c) were analyzed using one-way ANOVA for multiple-group comparisons, followed by Tukey’s two-tailed post-hoc test for pairwise analysis, all tests were two-sided. Source data are provided as a Source Data file.

To elucidate the potential antibacterial mechanisms of HIPCM, the following experiments were performed. First, the intracellular copper content was quantified, and results showed that the intracellular copper content of bacteria in biofilm increased dramatically following HIPCM treatment, indicating substantial intracellular copper influx (Fig. 6a). Cuproptosis, a copper-dependent cell death modality characterized by intracellular copper accumulation, destabilization of lipoylated enzymes, and fatal proteotoxic stress, has recently been documented in prokaryotes^39^, and this marked copper influx suggests that HIPCM may trigger a cuproptosis-like process. Enzymatic assays further demonstrated decreased activities of respiratory chain complexes I and II in both the HIPCM and the HIPCM + H_2_O_2_ groups (Fig. 6b, c), directly confirming respiratory chain disruption.

We then conducted transcriptomic profiling of *MRSA* treated with PBS (control) or HIPCM + H_2_O_2_, identifying 969 differentially expressed genes (DEGs), including 516 upregulated and 453 downregulated genes (Fig. 6d). A heatmap was used to visualize gene expression differences between the control and H_2_O_2_ + HIPCM groups, confirming the consistency of gene expression changes induced by H_2_O_2_ + HIPCM treatment (Fig. 6e). Gene ontology (GO) enrichment analysis highlighted terms related to transporter activity, metabolic processes, antioxidant regulation, and electron transport, indicating that HIPCM disrupts core pathways associated with ROS metabolism and redox buffering (Fig. 6f). Kyoto Encyclopedia of Genes and Genomes (KEGG) enrichment pathway analysis and heatmap visualization (Fig. 6g) revealed significant enrichment of pathways involved in ABC transporters, the TCA cycle, oxidative phosphorylation, virulence, and quorum sensing. Notably, HIPCM treatment markedly downregulated genes linked to oxidative phosphorylation and the cellular oxidative stress response, consistent with membrane integrity disruption, GSH depletion, and subsequent ROS overaccumulation. Concomitantly, transcription of genes involved in the TCA cycle, glyoxylate cycle, and pyruvate metabolism was significantly suppressed, collectively suggesting that HIPCM triggers a cuproptosis-like process in *MRSA*. Importantly, the downregulation of glutamate and aspartate metabolism genes implies impaired glutathione biosynthesis capacity, providing a transcriptomic explanation for HIPCM-induced GSH exhaustion and ROS amplification. Additionally, impaired ABC transporter function was observed, indicating diminished active transport and copper efflux capacity, thereby reinforcing the sustained metal accumulation required for cuproptosis. Furthermore, significant enrichment of genes related to alanine, aspartate, and glutamate metabolism was noted, suggesting potential effects on antimicrobial defense, oxidative stress adaptation, and cell wall synthesis. Finally, expression of quorum-sensing and virulence genes was suppressed.

Collectively, these results indicate that HIPCM exerts antibacterial activity through a coordinated mechanism involving GSH depletion, ROS accumulation, and TCA cycle disruption. These effects synergistically disrupt membrane integrity, interfere with electron transport, reduce ATP production, impair material transport, inhibit cell wall synthesis, and attenuate virulence, ultimately leading to bacterial death (Fig. 6h).

### HIPCM alleviates oxidative stress, enhances angiogenesis, and promotes fibroblast migration in vitro

**Fig. 7:**
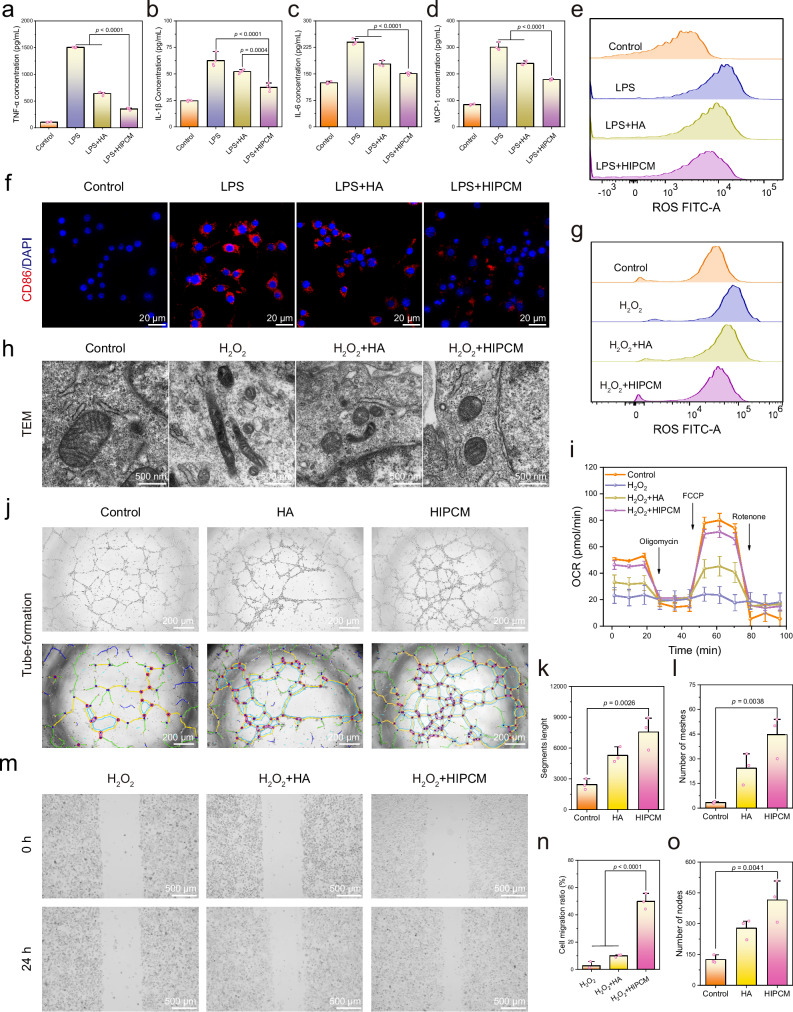
HIPCM alleviates oxidative stress, promotes cell migration, and enhances angiogenesis in vitro. **a-d** Relative levels of proinflammatory markers, including **a** TNF-α, **b** IL-1β, **c** IL−6, and **d** MCP−1, were quantified (*n* = 4 independent replicates). **e** Intracellular ROS levels in RAW 264.7 cells. **f** Immunofluorescence staining images of M1 (CD86) macrophages (*n* = 3 independent replicates; Scale bars, 20 μm). **g** Intracellular ROS levels in HUVECs. **h** TEM image depicting the mitochondrial ultrastructure in HUVECs (*n* = 3 independent replicates; Scale bars, 500 nm). **i** OCRs measurements (*n* = 3 independent replicates, data are presented as mean values ± SD). **j** Tube-formation assay (Scale bars, 200 μm). **k** Tube-forming segment length (*n* = 3 independent replicates). **l** Tube-forming meshes (*n* = 3 independent replicates). **m** Cell scratch-migration assay (Scale bars, 500 μm) and **n** quantitative migration ratio of L929 cells (*n* = 3 independent replicates). **o** Tube-forming branch nodes (*n* = 3 independent replicates). All data are expressed as the mean values ± SD. Statistical significance was determined using one-way ANOVA for multiple-group comparisons, followed by Tukey’s two-tailed post-hoc test for pairwise analysis, all tests were two-sided. Source data are provided as a Source Data file.

Excessive ROS and dysregulated inflammation following antibacterial therapy disrupt the coordinated functions of diverse cellular populations, including endothelial cells, macrophages, and fibroblasts, whose indispensable, synergistic functions are required for cell survival, migration, angiogenesis, and ultimately the successful wound regeneration^40^. To elucidate the bioactivity of HIPCM across key cell types involved in wound repair, we systematically evaluated its effects on RAW 264.7 inflammatory responses, endothelial oxidative injury and angiogenic function, and fibroblast migration. As shown in Fig. 7a–d, LPS stimulation elicited a pronounced increase in M1 macrophage-associated proinflammatory cytokines, including tumor necrosis factor-α (TNF-α), interleukin−1β (IL-1β), interleukin-6 (IL-6), and monocyte chemoattractant protein−1 (MCP−1), confirming the successful induction of M1 macrophage polarization. In contrast, HIPCM treatment significantly attenuated this cytokine cascade. Furthermore, given the central role of ROS as key messengers in amplifying inflammatory signaling^41^, flow cytometric analysis demonstrated that HIPCM markedly blunted the LPS induced intracellular ROS burst (Fig. 7e), an effect that likely contributes to its cytokine-suppressive activity. Complementary immunofluorescence imaging revealed that the LPS-driven increase in CD86 fluorescence, a marker of macrophage activation, was substantially reduced by HIPCM treatment (Fig. 7f). Together, these data indicate that HIPCM restores intracellular redox homeostasis and mitigates M1 macrophage-driven inflammatory responses, which is conducive to inflammation resolution during wound healing.

Next, we assessed whether HIPCM protects endothelial cells from oxidative injury while preserving their proangiogenic capacity using an H_2_O_2_-induced injury model in HUVECs. Flow cytometry with the ROS probe DCFH-DA confirmed that HIPCM pretreatment markedly reduced the H_2_O_2_-triggered increase in intracellular ROS (Fig. 7g), suggesting that HIPCM directly scavenges excess ROS. Notably, examination of mitochondrial morphology revealed that HIPCM mitigated H_2_O_2_-induced mitochondrial abnormalities, including shrinkage, swelling, cristae loss, and vacuolization (Fig. 7h and Supplementary Fig. 8). These ROS scavenging and mitochondria-protective effects are likely attributable to the antioxidant components of HIPCM. Given the tight coupling between cellular metabolism and endothelial angiogenic performance^42^, we quantified the effect of HIPCM on the oxygen consumption rates (OCRs) of endothelial cells under H_2_O_2_ induced oxidative stress. As shown in Fig. 7i, HIPCM treatment effectively alleviated the deleterious impact of elevated ROS on cellular respiration and energy metabolism, resulting in significant increases in basal respiration and maximal respiratory rate (Supplementary Figs. 9, 10). Functionally, a tube formation assay was performed to assess angiogenic capacity. As shown in Fig. 7j–l and o, HIPCM treatment resulted in longer capillary lengths and more branch nodes and meshes compared with the control. Taken together, these findings indicate that HIPCM can rescue HUVEC survival and function by improving the high ROS environment, thereby promoting angiogenesis and accelerating wound healing.

Finally, we examined the impact of HIPCM on fibroblast motility under oxidative stress. Compared with H_2_O_2_ alone, HIPCM increased L929 fibroblast migration by ~47% after 24 h of treatment (Fig. 7m, n). These findings suggest that HIPCM promotes physiological wound healing.

Taken together, these findings demonstrate that HIPCM suppresses ROS overproduction, dampens M1 macrophage-mediated inflammation, preserves endothelial mitochondrial function and angiogenesis, and regulates fibroblast migration to promote tissue repair. These findings support a multimodal mechanism by which HIPCM accelerates wound healing.

### Therapeutic efficacy of HIPCM in a *MRSA*-infected full-thickness skin defect model

**Fig. 8:**
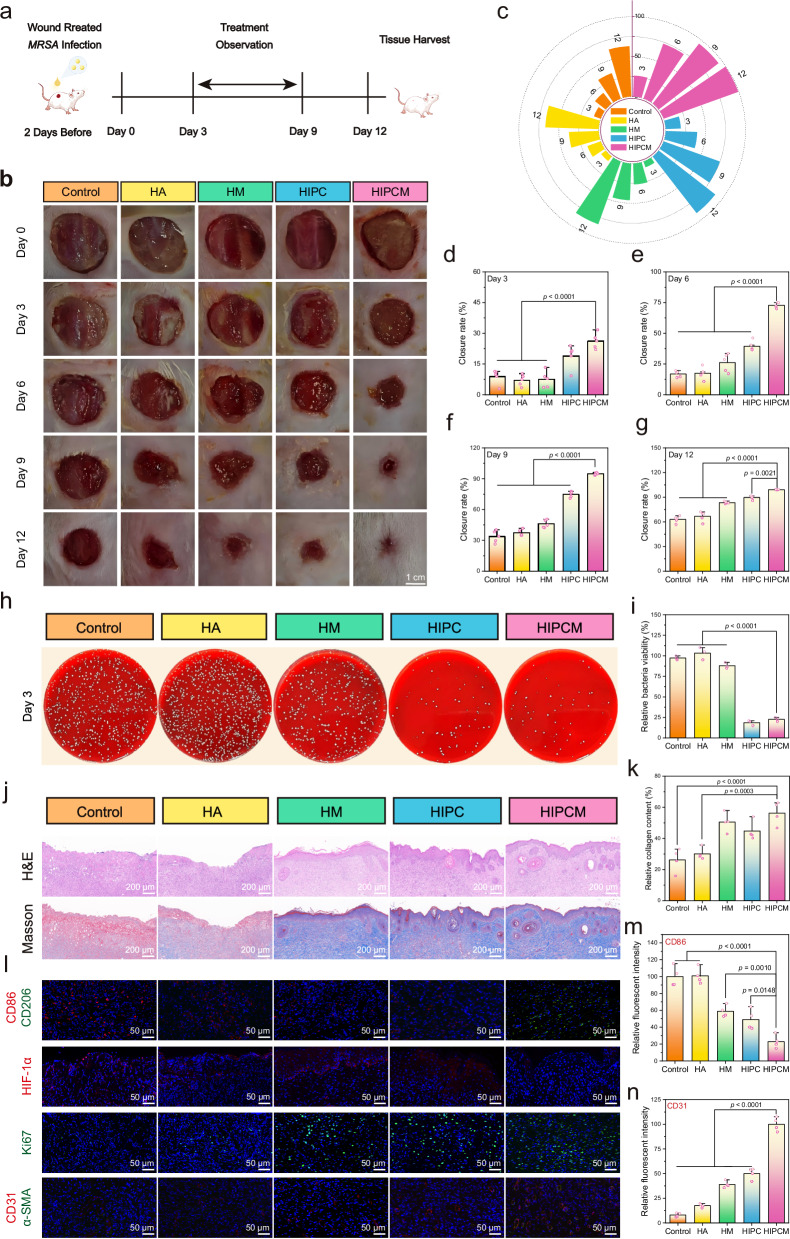
Therapeutic efficacy of HIPCM in a *MRSA*-infected full-thickness skin defect model. **a** Schematic representation of the *MRSA*-infected wound mouse model and treatment regimen. **b** Representative images of the wound healing process. **c** Wound healing rates under different treatments (Scale bars, 1 cm). **d–g** Quantitative analysis of wound healing rates at **d** day 3, **e** day 6, **f** day 9, and **g** day 12 (*n* = 5 biologically independent mice per group). **h** Images of surviving bacterial colonies in the focal area on day 3 and **i** the quantitative inhibition rate (*n* = 3 independent replicates). **j** Representative H&E and Masson staining images of wound tissues (Scale bars, 200 μm). **k** Quantitative analysis of collagen deposition (*n* = 4 independent replicates). **l** Immunofluorescence images of CD86 (red)/CD206 (green), HIF−1α (red), Ki67 (green), and CD31 (red)/α-SMA (green) expression (Scale bars, 50 μm). **m** Relative fluorescent intensity of CD86 expression (*n* = 4 independent replicates). **n** Relative fluorescent intensity of CD31 expression (*n* = 4 independent replicates). All data are expressed as the mean values ± SD. Statistical significance was determined using one-way ANOVA for multiple-group comparisons, followed by Tukey’s two-tailed post-hoc test for pairwise analysis, all tests were two-sided. Source data are provided as a Source Data file.

Building on HIPCM’s established regulatory effects, which include combating bacterial infection, suppressing inflammation, and promoting regenerative processes, we established a *MRSA*-infected full-thickness skin defect model to further evaluate its therapeutic efficacy in wound healing (Fig. 8a). One day after *MRSA* infection, mice were randomly divided to five treatment groups: PBS (control), HA gel, HM (HA + MA), HIPC (HA + IrPtCu), and HIPCM (HA + IrPtCu + MA). Serial imaging (Fig. 8b) and planimetric analysis (Fig. 8c–g) showed that HM, HIPC, and HIPCM significantly accelerated wound closure compared with PBS and HA gel. HIPCM exerted the most prominent effect, achieving ~90% closure by day 9 and near-complete healing by day 12. Antibacterial efficacy was verified by quantitative bacterial culture on day 3, which revealed markedly reduced bacterial loads in the HIPC and HIPCM groups, approaching eradication (Fig. 8h, i).

Histological observations were consistent with the macroscopic wound healing outcomes. Specifically, H&E staining demonstrated more intact tissue architecture and robust granulation tissue formation in the HIPC group, particularly in the HIPCM group, compared with the PBS and HA groups (Fig. 8j). Masson’s trichrome staining further revealed increased collagen deposition and improved fiber organization in the HM and HIPCM groups relative to the control group (Fig. 8k), with HIPCM-treated mice exhibiting the highest collagen density and most orderly fiber alignment. Immunofluorescence analysis of macrophage phenotypes (Fig. 8l) revealed the regulatory role of HIPCM in shaping the wound immune microenvironment. Compared with the other treatments, HIPC and HIPCM not only reduced the expression of the M1 macrophage marker CD86 but also induced a phenotypic shift toward the pro-healing M2 subtype. In contrast, HM and HIPC alone had no significant effect on the expression of either the M2 marker CD206, highlighting the immunoregulatory effect under the combined action of IrPtCu nanozyme and MA (Fig. 8m). Consistent with the normalized immune microenvironment, HIPC and HIPCM further improved local wound pathophysiology: they downregulated the expression of hypoxia inducible factor-1α (HIF-1α), a canonical marker of tissue hypoxia, indicating improved local oxygenation, which is a prerequisite for resolving inflammation and sustaining tissue repair^43^. Regarding cellular proliferation, Ki67 staining showed that hydrogel-based interventions generally enhanced cell proliferation across all groups; among these, HIPCM exerted the most potent effects in normalizing the infected wound microenvironment, likely due to their synergistic antibacterial, anti-inflammatory, and oxygenation promoting properties. Notably, the HIPCM group exhibited the strongest proangiogenic capacity, a critical determinant of nutrient delivery to the healing wound bed^44^. Co-immunofluorescence staining for α-smooth muscle actin (α-SMA) and cluster of differentiation 31 (CD31) (Fig. 8n) further confirmed these findings: compared with controls, HIPCM treatment markedly enhanced angiogenesis, underscoring its superior capacity to drive neovessel maturation and stabilization.

To evaluate their biocompatibility of HIPCM for in vivo therapeutic applications, histological analysis of the heart, liver, spleen, lung, and kidney was performed after 12 days of treatment, with no significant tissue abnormalities observed (Supplementary Fig. 11). In addition, there were no significant differences in physiological blood parameters among the groups, indicating favorable in vivo biocompatibility of HIPCM (Supplementary Fig. 11). Collectively, these results demonstrate that HIPCM can serve as a high-performance antibacterial wound treatment material.

### RNA sequencing reveals the therapeutic mechanisms of HIPCM

**Fig. 9:**
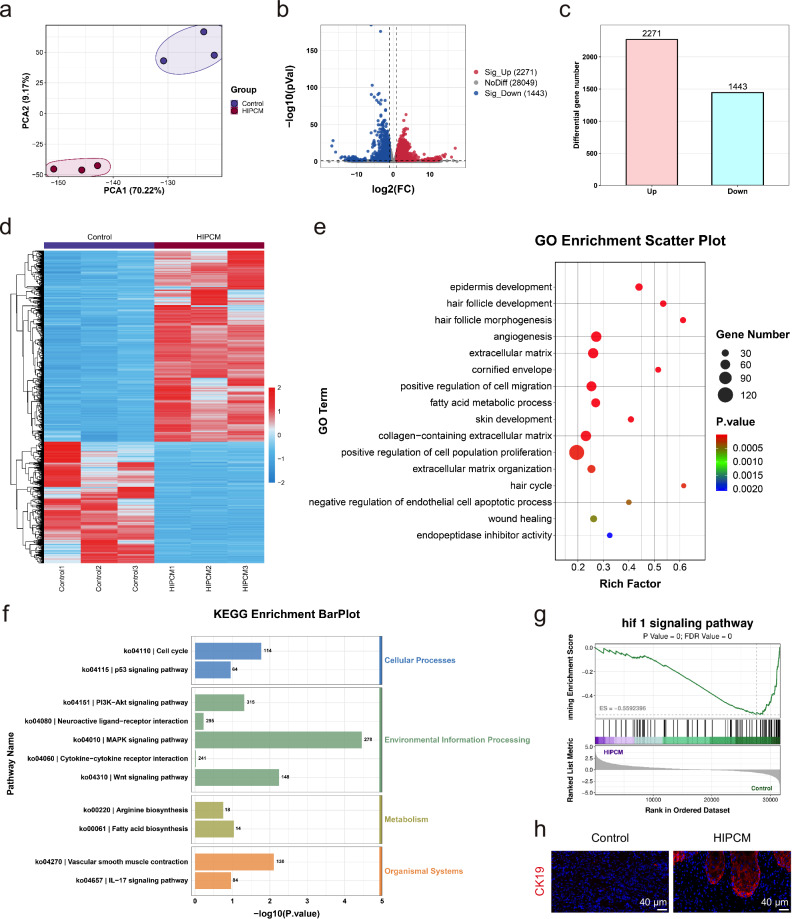
The therapeutic mechanisms of HIPCM as revealed by RNA sequencing. **a** PCA of the transcriptomic profiles. **b** Volcano plots comparing gene expression between the HIPCM and control groups **c** Number of DEGs. **d** Heatmaps of the screened differentially expressed genes involved in the healing process. **e** KEGG enrichment and **f** GO enrichment analysis of the DEGs. **g** GSEA of HIF−1 signaling pathway. **h** Immunofluorescence images of CK19 (*n* = 3 independent replicates; Scale bars, 40 μm). In a-h, the Control group indicates PBS. Experiments were repeated independently three times with similar results. In **e**, **f** *p* value obtained from one-sided Hypergeometric test without multiple comparisons. In g, *p* value obtained from two-sided, rank-based permutation test, with significance determined by FDR adjustment for multiple comparisons.

To elucidate the mechanisms underlying HIPCM-mediated wound healing, we collected wound tissues from mice in the control and HIPCM groups on day 12, followed by transcriptomic analysis. Principal component analysis (PCA) revealed significant differences in gene expression profiles between the HIPCM and control groups (Fig. 9a). A total of 3714 DEGs were identified following HIPCM treatment compared with the PBS (control) group (|log_2_FC | > 1, padj <0.05), of which 2271 were upregulated and 1443 were downregulated (Fig. 9b, c). A heatmap of the upregulated and downregulated DEGs is presented in Fig. 9d. Compared with the PBS group, GO enrichment analysis revealed pronounced upregulation of gene clusters involved in wound healing. Consistently, HIPCM treatment activated pathways related to angiogenic signaling and hair follicle morphogenesis, indicating a coordinated molecular program that promotes tissue regeneration (Fig. 9e). KEGG pathway analysis further revealed that HIPCM treatment upregulated multiple signaling cascades, including PI3K-Akt, cytokine-cytokine receptor interaction, cell cycle, ECM receptor interaction, MAPK and Wnt signaling (Fig. 9f). In addition, GSEA demonstrated that HIF-1α signaling was significantly downregulated following HIPCM treatment (Fig. 9g), which is consistent with our previous immunofluorescence results.

On the basis of the above results, we postulate that HIPCM not only accelerates epithelial regeneration and wound closure but also facilitates the reconstruction of hair follicles and other adnexal structures. Cytokeratin 19 (CK19) is recognized as a definitive marker of hair follicle stem cells and is widely regarded as an indicator of their regenerative potential within follicular tissue^45^. Furthermore, immunofluorescence images showed well-defined hair follicle structures with robust CK19 expression following HIPCM treatment (Fig. 9h). Overall, the therapeutic efficacy of HIPCM is primarily attributable to its capacity to promote cellular proliferation, differentiation, and directional migration; regulate cell cycle progression; and orchestrate tissue repair and regeneration, thereby eliciting a coordinated response that significantly accelerates the healing of *MRSA*-infected wounds and supports the restoration of skin architecture and function.

### Therapeutic efficacy of HIPCM in Bama mini-pigs wound healing

**Fig. 10:**
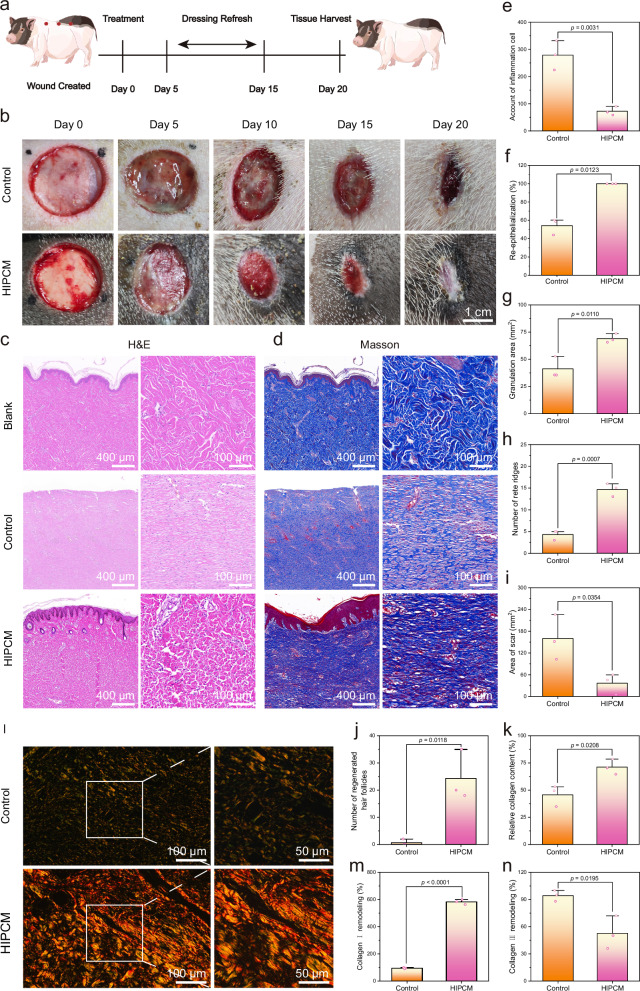
Therapeutic efficacy of HIPCM in Bama mini-pig wound healing. **a** Schematic representation of the full-thickness skin defect model established in Bama mini-pig and the treatment regimen. **b** Representative images of the wound healing process in Bama mini-pig model (Scale bars, 1 cm). **c** Representative H&E and **d** Masson’s trichrome staining images of wound tissues (Scale bars, 400 μm and 100 μm). **e–k** Quantification of **e** inflammatory cells, **f** re-epithelialization, **g** granulation area, **h** number of rete ridges, **i** scar area, **j** number of regenerated hair follicles and **k** relative college content (*n* = 3 independent replicates). **l** Sirius Red-stained in the center of the repaired tissue (green fluorescence indicates type III collagen, and red fluorescence represents type I collagen, scale bars, 100 μm and 50 μm). **m, n** Quantitative statistics of **m** type I collagen and **n** type III collagen (*n* = 3 independent replicates). All data are expressed as the mean values ± SD. Statistical significance was determined using the two-tailed Student’s t-test for two-group comparisons. Source data are provided as a Source Data file.

To further evaluate the therapeutic efficacy and clinical translatability of HIPCM beyond the *MRSA*-infected wound model, we established a full-thickness skin defect model in Bama mini-pigs (Fig. 10a), whose skin shares high structural and physiological homology with human skin, such as comparable epidermal thickness, hair follicle density, collagen organization, and wound-healing patterns, thereby enhancing the clinical relevance of the study^46,47^. Bama mini-pigs received either PBS or HIPCM treatment, and wound healing was monitored by serial planimetric analysis at day 5, day 10, day 15, and day 20. Compared with control wounds, HIPCM-treated wounds exhibited a significantly faster closure rate (Fig. 10b and Supplementary Fig. 12). In addition, wound healing outcomes were further confirmed by H&E staining (Fig. 10c) and Masson’s trichrome staining (Fig. 10d). H&E staining revealed that HIPCM substantially reduced inflammatory cell infiltration (Fig. 10e), indicating potent anti‑inflammatory activity at the wound site. This early resolution of inflammation likely laid the foundation for the timely initiation of the proliferative phase, as evidenced by enhanced re-epithelialization (Fig. 10f) and increased formation of newly generated granulation tissue (Fig. 10g). The regenerated tissue exhibited more pronounced rete ridge structures (Fig. 10h) and a higher density of nascent hair follicles (Fig. 10j), indicating the restoration of skin appendages and a closer resemblance to native skin architecture. Additionally, Masson’s trichrome staining revealed enhanced collagen fiber formation in HIPCM-treated wounds, indicating favorable wound healing outcomes (Fig. 10k). Moreover, Sirius Red-stained under polarized light (Fig. 10l) revealed a collagen profile skewed toward mature type I collagen, with reduced immature type III collagen (Fig. 10m, n), as well as fiber organization approaching that of uninjured skin (Supplementary Fig. 13). HIPCM-treated wounds exhibited marked attenuation of scar formation (Fig. 10i). This shift not only reflects enhanced tissue maturity but also suggests improved mechanical integrity and elasticity of the regenerated skin.

Collectively, in a clinically relevant large‑animal model, HIPCM accelerates wound healing in Bama mini-pigs through a dual sequential mechanism: early and effective suppression of inflammation, followed by promotion of organized tissue regeneration. The reduced inflammatory burden likely minimizes fibroblast overactivation and excessive scar formation, whereas the promotion of mature collagen assembly and skin appendage regeneration enhances long term functional restoration. These findings establish HIPCM as a clinically translatable, comprehensive wound management strategy applicable to a broad spectrum of scenarios, including but not limited to infected wounds.

## Discussion

Effective therapeutic management of drug‑resistant bacterium‑infected wounds remains one of the most challenging tasks in modern clinical medicine^1,2^. Current antibacterial strategies primarily rely on systemic antibiotics and topical antibacterial dressings, which often fail to achieve satisfactory long‑term outcomes due to poor adaptability to the dynamic wound microenvironment and the rising prevalence of multidrug‑resistant pathogens^48^. Moreover, most existing treatments emphasize single‑stage antibacterial activity without addressing subsequent inflammatory resolution and tissue regeneration, leading to delayed wound closure and non‑functional repair. Therefore, a microenvironment adaptive therapeutic material capable of sequentially regulating the wound‑healing process is crucial for overcoming these clinical challenges^49^. In this study, we developed a multifunctional hydrogel platform that integrates an IrPtCu multimetallic nanozyme with MA to achieve sequential antibacterial, anti‑inflammatory, and pro‑regenerative effects tailored to the evolving wound milieu.

The central feature of HIPCM lies in the rationally engineered IrPtCu nanozyme, which functions as a pH-responsive Janus catalyst. Unlike conventional unimodal nanozymes, which often suffer from uncontrolled ROS production and subsequent off-target tissue damage, HIPCM achieves a better balance between pathogen eradication and tissue protection. Mechanistically, our DFT calculations and kinetic analyses reveal that the incorporation of Ir into the PtCu lattice fundamentally optimizes the electronic structure of the catalytic surface. Specifically, Pt provides the primary catalytic sites for ROS reactions^50^, Cu contributes to targeted antibacterial interactions^51^, and and Ir facilitates the adsorption and activation of H_2_O_2_ and simultaneously catalyzes the efficient oxidation of GSH to GSSG^52^. The balance among these three elements collectively modulates its multi-enzyme activities. This synergistic alloying effect profoundly reduces the energy barrier for H_2_O_2_ activation and ·OH release. This design endowed HIPCM with dual catalytic reactivity and a Janus-like behavior, allowing both ROS generation and scavenging depending on the local pH. Under acidic infection‑mimicking conditions, the nanozyme exhibited enhanced peroxidase‑ and oxidase‑like activities, efficiently decomposing H_2_O_2_ and O_2_ into bactericidal ROS, while simultaneously depleting intracellular GSH to weaken bacterial antioxidant defense. When the microenvironment returned to neutral pH following infection control, IrPtCu displayed strong SOD‑ and CAT‑like activities, scavenging superoxide and hydroxyl radicals to prevent oxidative injury to host cells. This pH‑responsive, bidirectional catalysis differentiates HIPCM from conventional unimodal nanozymes, enabling spatiotemporal control of oxidative stress throughout the healing cascade.

Transitioning from catalytic mechanisms to biological outcomes, the multifactorial antibacterial profile of HIPCM offers a robust strategy to circumvent the escalating threat of antimicrobial resistance. Transcriptomic and metabolic analyses underscore that HIPCM triggers a systemic metabolic collapse in *MRSA*. The concurrent induction of membrane disruption, ROS/GSH disequilibrium, and notably, copper-induced cuproptosis-like death, constitutes a multifaceted lethal stress for bacteria^53^. This indicates that targeting bacterial metabolic vulnerabilities alongside traditional ROS-mediated damage can effectively bypass the adaptive resistance mechanisms commonly associated with conventional antibiotics. Furthermore, the therapeutic mandate of HIPCM extends far beyond microbial eradication. By actively remodeling the inflammatory and oxidative microenvironments, HIPCM facilitates the critical biological transition from the inflammatory phase to the proliferative phase. The observed suppression of M1 macrophage polarization, alongside the restoration of endothelial mitochondrial function and the promotion of fibroblast migration under oxidative stress, suggests that HIPCM acts as a comprehensive microenvironment modulator rather than a mere antimicrobial agent. Ultimately, these findings highlight a promising paradigm shift in treating infected wounds: moving from solely eradicating pathogens to simultaneously rescuing local cellular metabolism and orchestrating the entire regenerative cascade.

The robust mechanistic foundation established in vitro translates seamlessly into profound in vivo therapeutic efficacy, as evidenced across both *MRSA*-infected murine models and clinically relevant Bama mini-pig full-thickness defect models. Rather than merely accelerating macroscopic wound closure, HIPCM orchestrates a structural and functional restoration of the skin. This effective stage-adaptive repair underscores a critical synergistic interplay between the Janus nanozyme and natural herbal medicines: where nanozyme-mediated oxidative stress regulation, cooperating with MA facilitated ordered collagen deposition and angiogenesis to achieve stage-adaptive tissue repair, accompanied by improved collagen alignment, angiogenesis, and M2 macrophage polarization, all collectively contributing to accelerated and high‑quality tissue regeneration. Transcriptomic analysis of HIPCM‑treated wounds highlighted the activation of multiple regenerative signaling pathways, including PI3K‑Akt, MAPK, Wnt, and TGF‑β networks, alongside downregulation of HIF‑1α signaling, reaffirming improved oxygenation and redox homeostasis. Importantly, the presence of robust CK19‑positive hair follicles in regenerated tissues indicated recovery of skin appendages and functional renewal, such a high degree of tissue complexity is rarely achieved by traditional antimicrobial strategies and highlights the necessity of active, multi-target microenvironment modulation. Importantly, the use of Bama mini-pig models, whose skin structure and healing processes closely resemble those of humans, provided a highly representative platform for preclinical evaluation. The successful regeneration of full-thickness wounds in this large-animal model highlights the scalability and clinical relevance of HIPCM therapy^46,47^, underscoring its strong potential for translation into advanced wound management and reconstructive applications.

In conclusion, HIPCM constitutes a microenvironment‑adaptive therapeutic system that integrates rational nanozyme catalysis and bioactive hydrogel chemistry to coordinate antibacterial defense, inflammatory resolution, and regenerative healing throughout the entire wound‑healing process. We demonstrated that by employing a pH‑responsive IrPtCu Janus nanozyme combined with MA‑mediated signaling, HIPCM transforms the hostile, infected wound microenvironment into one favorable for regeneration, thereby achieving efficient disinfection, rapid inflammation resolution, and functional tissue recovery. Therefore, the developed hydrogel platform offers a robust strategy for microenvironment regulation and the synergistic application of natural herbal medicines, providing a comprehensive and clinically translatable hydrogel platform for the management of infected and other complex wounds.

## Methods

### Ethics approval

All the animal experiments were conducted in compliance with the Chinese National Standard and under the protocols that were approved by the Animal Ethics Committee of the Academy of Military Medical Sciences (IACUC – DWZX – 2024 – P536).

### Materials

All chemicals and solvents were commercially available and used without further purification. Iridium trichloride (IrCl_3_) was purchased by Beijing Innochem Science & Technology Co., Ltd. (China). Chloroplatinic acid hexahydrate (H_2_PtCl_6_·6H_2_O), copper chloride (CuCl_2_), L-ascorbic acid, Hyaluronic acid (HA-T), 1,4-butanediyl diglycidyl ether (BDDE), 3,3’,5,5’-tetramethylbenzidine (TMB) 5,5’-dithiobis-(2-nitrobenzoic acid) (DTNB), 1,1-diphenyl-2-picrylhydrazyl (DPPH), 2, 2′-azino-bis (3-ethylbenzothiazoline-6-sulfonic acid) (ABTS), 2-phenyl-4,4,5,5-tetramethylimidazoline-3-oxide-1-oxyl (PTIO), and ferrous sulfate (Fe_2_SO_4_) were provided by Aladdin Chemical Reagent Co., Ltd. (China). Madecassoside was purchased by Shanghai Macklin Biochemical Co., Ltd. (China). DMPO, TEMPO, xanthine oxidase, and hypoxanthine were purchased by MedChemExpress LLC Co., Ltd. (USA). ELISA kits of TNF-α, IL-1β, MCP-1and IL-6, and BCA protein colorimetric assay kit, were purchased by Wuhan Elabscience Biotechnology Co., Ltd. (China). Live/Dead bacteria staining kit and 2’,7’-dichlorofluorescin diacetate were purchased by Beijing Solarbio Science & Technology Co., Ltd. (China). Anti-CD86 (Rabbit mAb), anti-CD206 (Rabbit mAb), anti-alpha smooth muscle (Rabbit pAb), anti-CD31 (Mouse mAb), anti-HIF-1 alpha (Rabbit mAb), anti-Ki67 (Mouse mAb), and anti-CK19 (Rabbit mAb) were purchased by Wuhan Servicebio Technology Co., Ltd. (China).

### Synthesis of IrPtCu and PtCu nanozymes

0.75 mL of 10 mM IrCl_3_, 2.25 mL of 10 mM H_2_PtCl_6_·6H₂O and 3.0 mL of 10 mM CuCl_2_ were sequentially introduced into 4.0 mL DI water maintained at 40 °C under continuous magnetic stirring, yielding a homogeneous light green transparent solution. Subsequently, 3 mL of 100 mM L-ascorbic acid solution was added, leading to an immediate color change to gray, which progressively darkened during the reaction. The mixture was allowed to react for 4 h to ensure complete reduction. The resulting dispersion was collected by centrifugation (8000 × *g*), washed with DI water three times to remove residual species, and stored for further characterization. For the PtCu control nanozyme, synthesis was performed under identical conditions without the addition of IrCl_3_.

### Synthesis of the HIPCM

IrPtCu nanozyme (5 mg) was dispersed in 0.99 mL DI water and sonicated for 3 min to achieve uniform dispersion. Sodium hydroxide (10 mg) was then dissolved in the suspension, followed by the addition of 10 μL 1,4‑butanediol diglycidyl ether under continuous stirring. Subsequently, sodium hyaluronate (100 mg) was introduced, and the mixture was incubated at 37 °C for 4 h to form the hydrogel network. The resulting hydrogel was washed with 1 M HCl until the pH reached 7.4 and subjected to dialysis for 48 h to remove residual species. The purified hydrogel was freeze‑dried for 48 h to yield IrPtCu nanozyme hydrogel powder. For HIPCM preparation, madecassoside powder (10 mg) was blended with IrPtCu nanozyme hydrogel powder (100 mg) and reconstituted with 1 mL DI water.

### Materials characterization

The morphology and structure of IrPtCu nanozymes were characterized using a field-emission high-resolution transmission electron microscope (JEOL, JEM-F200, Japan). The morphology and structure of HIPCM were examined using a field-emission scanning electron microscope (Zeiss, Sigma 360, Germany). The elemental distribution in the IrPtCu nanozyme and HIPCM was characterized using energy-dispersive spectroscopy (EDS) mapping analysis. The elemental valence and chemical composition of the IrPtCu nanozyme were characterized using X-ray powder diffraction (Rigaku, SmartLab SE, Japan) and an X-ray photoelectron spectrometer (Thermo Scientific, K-Alpha, USA). The chemical structures were determined by Fourier transform infrared spectroscopy (Thermo Scientific, Nicolet iS20, USA) in the range of 400–4000 cm^−1^. A rotational rheometer (Kinexus Prime lab + , NETZSCH, Germany) was used to detect the storage (G′) and loss (G′′) moduli of the hydrogels at 37 °C, the frequency sweep was measured with a scan frequency from 0.1 to 10 rad/s. The viscosity was measured over a shear rate range of 0.1–100 s^−1^.

### Swelling Tests

The swelling behavior of the hydrogels was evaluated in phosphate‑buffered saline (PBS, pH 7.4) at 37 °C. Wet hydrogels were first weighed to determine the initial mass (W_0_). At predetermined time intervals, swollen hydrogels were removed from PBS, and excess surface water was gently blotted with filter paper. The mass of the swollen hydrogels (W_t_) was then measured. Swelling ratio = W_t_/W_0_ × 100%.

### Hemolytic assay

RBCs were obtained by centrifuging mouse blood at 600 × *g* for 10 min to remove the buffy coat. The RBC pellet was diluted in PBS to a final concentration of 1% (v/v) and washed twice with PBS. Hydrogel extracts (500 μg mL^−1^) were added to 900 µL of the RBC suspension in 1.5 mL microtubes. DI water served as the positive control, and PBS as the negative control. Samples were incubated at 37 °C for 2 h, followed by centrifugation at 600 × *g* for 10 min. Supernatants (200 μL) were transferred to a 96‑well plate, and absorbance at 540 nm was recorded using a microplate reader (Molecular Devices, SpectraMax iD5, USA). The hemolysis percentage was calculated based on the absorbance values relative to the positive and negative controls.

### Cytocompatibility evaluation

HUVECs were seeded in 96‑well culture plates at a density of 1 × 10^4^ cells mL^−1^ and allowed to adhere for 24 h. The cells were then treated with hydrogel extracts (500 μg mL^−1^) and evaluated at day 1, day 2, and day 3. Cell viability at each time point was assessed using the CCK‑8 assay according to the manufacturer’s instructions.

### DFT Calculations

DFT calculations were performed using the Vienna Ab‑initio Simulation Package (VASP) with the projector‑augmented wave method and the Perdew–Burke–Ernzerhof functional plus DFT‑D3 correction. A Pt(111) 5 × 5 × 1 supercell and Ir_83_Pt_17_(111) slab models (with 0, 2, and 4 OH groups) were constructed, with a 25 Å vacuum layer along the *c*‑axis. The plane‑wave cut‑off energy was 450 eV, and Brillouin‑zone sampling used a 2 × 2 × 1 Monkhorst grid. Structures were optimized until the energy converged to 10^−5 ^eV and forces were below 0.02 eV Å_-1_. Spin polarization was applied. Gibbs free energies were determined using the computational hydrogen electrode model, with zero‑point energy and entropy corrections at 298.15 K and 1 atm. Reaction steps followed the proposed POD pathway for H_2_O_2_ decomposition, and necessary thermodynamic corrections were obtained via vaspkit.

### Multi-enzyme activities of nanozymes

The multi-enzyme activities of the nanozyme were evaluated by established colorimetric or oxygen measurement assays, with POD- and GSH-Px-like were determined under nitrogen when applicable. POD- and OXD-like activities were determined using the TMB oxidation assay in NaOAc-HOAc buffer (pH 5.5), varying H_2_O_2_ or TMB concentrations, respectively; absorbance at 652 nm was recorded after defined incubation periods. GSH-Px- and GSH-Ox-like activities were assessed using the DTNB assay in NaOAc-HOAc buffer (pH 5.5), varying GSH and, for GSH-Px, H_2_O_2_ concentrations; absorbance at 412 nm was measured after the reaction. CAT-like activity was examined by monitoring dissolved oxygen in PBS (pH 7.4) during H_2_O_2_ decomposition. SOD-like activity was evaluated by the NBT assay in PBS (pH 7.4), varying xanthine concentrations in the presence of xanthine oxidase, with absorbance recorded at 560 nm. For all assays, nanozyme concentration was fixed at 60 μg mL^−1^. The Michaelis-Menten parameters, including the *V*_max_ and the *K*_m_, were obtained from the saturation curves according to standard steady-state enzyme kinetic methods.

### Free radical scavenging activities of nanozymes

1,1-diphenyl-2-picrylhydrazyl (DPPH), 2, 2′-azino-bis (3-ethylbenzothiazoline-6-sulfonic acid) (ABTS) and 2-Phenyl-4,4,5,5-tetramethylimidazoline-3-oxide-1-oxyl (PTIO) assays were carried out to evaluate the free radical scavenging ability.

### The ⋅OH scavenging capacity of nanozymes

The ability of the nanozyme to eliminate hydroxyl radicals was evaluated by electron spin resonance (ESR) using DMPO as the spin-trapping agent. In brief, PBS (20 μL), H_2_O_2_ (100 mM, 10 μL), DMPO (5 μL), and nanozyme dispersion (5 μL) were combined, followed by the addition of FeSO4 solution (10 mM, 10 μL) to initiate the reaction, giving a final volume of 50 μL. The resulting solution was transferred into a sealed glass capillary tube (50 μL, inner diameter 1 mm) and analyzed by ESR after incubation for 5 min. The DMPO/⋅OH adduct generated a typical four-line signal (1:2:2:1). Attenuation of this signal was taken to indicate reduced ⋅OH levels and thus hydroxyl radical scavenging by the nanozyme. ESR measurements were performed at a microwave power of 2 mW, modulation amplitude of 1 G, scan width of 100 G, and receiver gain of 20 dB.

### The ^1^O_2_ scavenging capacity of nanozymes

The ^1^O_2_ scavenging activity of the nanozyme was also assessed by ESR. PBS (20 μL), H_2_O_2_ (100 mM, 10 μL), TEMPO (5 μL), and nanozyme dispersion (5 μL) were first mixed, after which FeSO_4_ solution (10 mM, 10 μL) was introduced to start the reaction. The final mixture (50 μL) was loaded into a sealed glass capillary tube (50 μL, inner diameter 1 mm). ESR spectra were collected after 5 min. The TEMPO/^1^O_2_ adduct showed a characteristic triplet pattern (1:1:1), and a weakened signal indicated a lower ^1^O_2_ level in the system. Instrument parameters were identical to those used in the ⋅OH assay.

### In vitro antibacterial experiment

The antibacterial activity of the HIPCM was evaluated in vitro against *MRSA* (ATCC 43300) and *E. coli* (ATCC 29522) using the agar plate counting method. The bacterial suspensions were treated under four different conditions: *MRSA*/*E. coli* alone, *MRSA*/*E. coli* + H_2_O_2_, *MRSA*/*E. coli* + HIPCM, and *MRSA*/*E. coli* + H_2_O_2_ + HIPCM. The final concentrations of bacteria, H_2_O_2_, and HIPCM in the reaction systems were 1 × 10^6^ CFU mL^−1^, 100 μM, and 500 μg mL^−1^, respectively. The suspensions were then diluted, plated, and incubated for 16 h at 37 °C. Bacterial morphology after different treatments was observed using SEM (Zeiss, Sigma 360, Germany). Protein leakage in the supernatants was quantified using a BCA protein assay kit. For live/dead bacterial staining, the treated bacteria were incubated with SYTO-9 and PI at 37 °C for 20 min. Fluorescence images were acquired using CLSM, and the ratio of green to red fluorescence intensity was quantified using ImageJ 1.53.

Intracellular ROS generation was evaluated using the DCFH-DA probe. Briefly, the four groups of bacterial suspensions were incubated with DCFH-DA at 37 °C for 20 min. After incubation, the samples were washed three times with PBS, and the green fluorescence was detected using Confocal laser scanning microscope (Leica, STELLARIS 5, Germany).

### Biofilm Inhibition Assay

100 μL of *MRSA*/*E. coli* (1 × 10^8^ CFU mL^−1^) was added to PBS, H_2_O_2_, HIPCM, or H_2_O_2_ + HIPCM solutions and incubated at 37 °C for 48 h to allow mature biofilm formation. After incubation, the supernatant was removed, and the biofilms attached to the bottom of the wells were gently washed three times with PBS. The remaining biofilms were stained with crystal violet and imaged using optical microscopy. Subsequently, the stained biofilms were dissolved in 33% acetic acid, and the absorbance was measured at 570 nm using a microplate reader for quantitative analysis.

### RNA-seq analysis

*MRSA* was cocultured with PBS, H_2_O_2_ + HIPCM at 37 °C for 6 h. Following treatment, the bacteria were harvested for RNAseq analysis, conducted by Shanghai Personalbio Co., Ltd. on the Illumina sequencing platform. The raw transcriptome sequencing data were subjected to quality control (QC) to remove adapter sequences, low-quality reads, and non-target sequences before being used for subsequent analyses. Gene Ontology (http://www.geneontology.org) and KEGG were used to analyze the gene functions. Differential gene expression analysis was performed using the R package, and those genes conformed to |log2 Fold change | > 1 (*p*-value < 0.05) were considered to be DEGs.

### Mitochondrial Morphological Analysis

Mitochondrial morphological integrity was assessed following the Flameng method. Briefly, ultrathin sections of tissue samples were prepared and examined using TEM (Hitachi, HT7800, Japan). Mitochondrial ultrastructural alterations were evaluated and graded according to the extent of mitochondrial swelling, disruption of cristae, and compromise of membrane integrity, providing a quantitative measure of mitochondrial damage.

### Cell treatments

For the LPS-stimulated RAW 264.7 macrophage model, cells (8 × 10^5^ cells mL^−1^) were seeded in 6‑well plates and cultured for 24 h, followed by incubation with complete DMEM containing LPS (500 ng mL^−1^) and hydrogel extracts (500 μg mL^−1^) for another 24 h. The supernatants were collected, and inflammatory cytokines (IL‑1β, TNF‑α, IL‑6, and MCP‑1) were quantified using ELISA kits. For the H_2_O_2_-stimulated HUVECs model, cells (4 × 10^5^ cells mL^−1^) were seeded in 6‑well plates, cultured for 24 h, and then treated with complete DMEM containing H_2_O_2_ (200 μM) and hydrogel extracts (500 μg mL^−1^) for 4 h to induce oxidative stress.

### Flow cytometry

The cells were collected in serum-free medium containing 10 μmol L^−1^ DCFH‑DA, followed by incubation at 37 °C for 20 min in the dark. Subsequently, the cells were centrifuged, washed, and resuspended in PBS. Intracellular ROS levels were analyzed using Flow Cytometric Cell Sorter (Becton, Dickinson and Company, BD FACSAria^TM^ II, USA).

### Cell migration capacity

L929 cells were cultured by the culture insert for 24 h. Thereafter, cells were subsequently removed from the culture insert and treated with serum-free medium containing H_2_O_2_ (200 μM) and hydrogel extracts (500 μg mL^−1^). Cell migration was observed at different time points and quantified using ImageJ 1.53.

### *MRSA*-infected wound mouse model

Eight-week-old male ICR mice were selected for the *MRSA*-infected wound model. The mice were anesthetized with 1% pentobarbital sodium (40 mg kg^−1^), and the dorsal hair was shaved. A full-thickness excisional wound with a diameter of ~12 mm was created on the dorsal skin. Subsequently, 30 μL of *MRSA* suspension (1 × 10^9^ CFU mL^−1^) was inoculated onto the wound surface and allowed to air-dry. After 24 h, the mice were randomly divided into five treatment groups: PBS (control), HA gel, HM (HA + madecassoside), HIPC (HA + IrPtCu), and HIPCM (HA + IrPtCu + madecassoside). The corresponding formulations were applied to the *MRSA*-infected wounds. Digital images of the wounds were captured at predetermined time points to monitor wound healing. Wound areas were quantified using ImageJ 1.53. On day 12 post-treatment, mice were euthanized and skin tissue samples from the wound sites were harvested for histological analysis, including H&E staining and Masson’s trichrome staining.

### Biocompatibility evaluation

After 12 days of treatment, major organs (heart, liver, spleen, lungs, kidneys) were collected for H&E staining to assess systemic biocompatibility. Blood samples were obtained for plasma biochemical analysis (MNCHIP, Celercare V5, China) and complete blood count testing (Mindray Animal Care, BC-5000 Vet, China).

### Bama mini-pig wound model

Bama mini-pig with an average body weight of ~10 kg were used to establish a full-thickness skin wound model. Intramuscular ketamine anesthesia is changed to isoflurane respiratory anesthesia. After anesthesia, three circular wounds were created on each side of the dorsal region using a 2.5 cm diameter biopsy punch (six wounds per pig). The skin at each wound site was excised with a scalpel to generate full-thickness skin defects with a thickness of ~2–3 mm, exposing the underlying subcutaneous fat. The wounds were then randomly assigned to two treatment groups: PBS (control) and HIPCM. The corresponding treatments were applied to the wound surfaces and covered with 3 M sterile dressings to prevent displacement. Digital photographs of the wounds were taken at designated time points to monitor wound healing. On day 20 post-treatment, the pigs were euthanized, and skin tissue samples from the wound sites were harvested for histological evaluation. The collected samples were subjected to H&E staining, Masson’s trichrome staining, and Sirius Red staining to assess tissue regeneration and collagen deposition.

### Statistical analysis

Data analysis was conducted using OriginPro 2024b, ImageJ 1.53 was utilized for the quantitative analysis of in vitro and in vivo imaging. Quantitative data were collected at least three independent times. All data are expressed as the mean values ± SD. Statistical significance was determined using the Student’s t-test for two-group comparisons, and one-way ANOVA for multiple-group comparisons, followed by Tukey’s two-tailed post-hoc test for pairwise analysis, all tests were two-sided.

### Reporting summary

Further information on research design is available in the Nature Portfolio Reporting Summary linked to this article.

## Supplementary information

Supplementary Information

reporting summary

Transparent Peer Review file

## Source data

source data

## Data Availability

All raw data generated for the figures in this study are provided in the source data file. The raw sequencing data generated in this study have been deposited in the Genome Sequence Archive (GSA; Genomics, Proteomics & Bioinformatics, 2025) in National Genomics Data Center (NGDC; Nucleic Acids Research, 2025), China National Center for Bioinformation, Chinese Academy of Sciences, under accession numbers CRA038796 and CRA038863. Source data are provided with this paper.
