# Supplementary material for: Self-adaptive nanozymes with enhanced multi-enzyme activities for sequential multimodal therapy of drug-resistant bacteria-infected wounds
Source: Nat Commun. 2026 May 28;17:6935. doi: 10.1038/s41467-026-73672-2 (PMC13389173; doi:10.1038/s41467-026-73672-2)
Supplement: Supplementary file 1 — Supplementary Information [file 41467_2026_73672_MOESM1_ESM.pdf]

Supplementary Information

Self-Adaptive Nanozymes with Enhanced Multi-Enzyme Activities for Sequential Multimodal Therapy of Drug-Resistant Bacteria-Infected Wounds

Xiaoyong Zhang[1], Hang Yu[1], Kai Zhu[1], Yao Xiao[1], Yuxuan Gong[1], Dandan Che[1], Wanyi Chen[1], Guoxing You[1], Xiyun Yan[2], Quan Wang[1], Kelong Fan[2]*, Hong Zhou[1]*, Gan Chen[1]**

[1]Academy of Military Medical Sciences, Beijing 100850, China

[2]CAS Engineering Laboratory for Nanozyme, Key Laboratory of Biomacromolecules (CAS), CAS Center for Excellence in Biomacromolecules, Institute of Biophysics, Chinese Academy of Sciences, Beijing 100101, China

*Correspondence to:

Quan Wang, wangquan0220@126.com;

Kelong Fan, fankelong@ibp.ac.cn;

Hong Zhou, zhouhtt1966@163.com;

Gan Chen, chenlzu2005@163.com.

This PDF file includes:

Supplementary Table

Supplementary Figures

Supplementary Table:

	Ir<br>mg·L^{-1}	Pt<br>mg·L^{-1}	Cu<br>mg·L^{-1}
IrPtCu	71.13	59.68	63.51

Supplementary Table 1. ICP-OES results of the contents of Ir, Pt, and Cu in IrPtCu nanozyme.

Supplementary Figures:

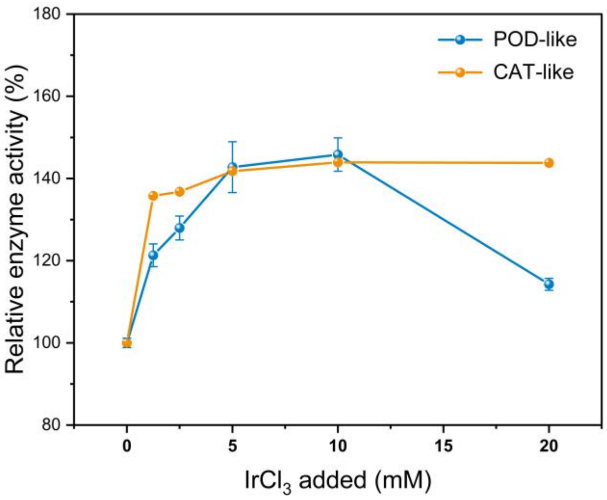

Supplementary Fig. 1. Effect of different $IrCl_3$ addition concentrations on the POD- and CAT-like activities of IrPtCu nanozyme (n = 4 independent replicates, data are presented as mean values ± SD). Source data are provided as a Source Data file.

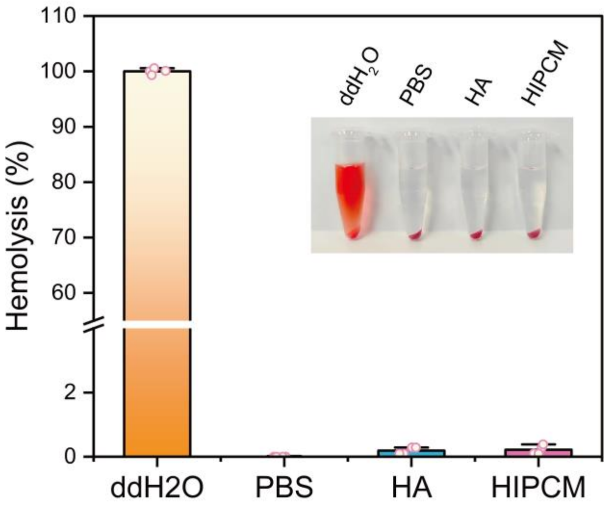

Supplementary Fig. 2. Hemolysis ratio. (n = 4 independent replicates, data are

presented as mean values ± SD) Source data are provided as a Source Data file.

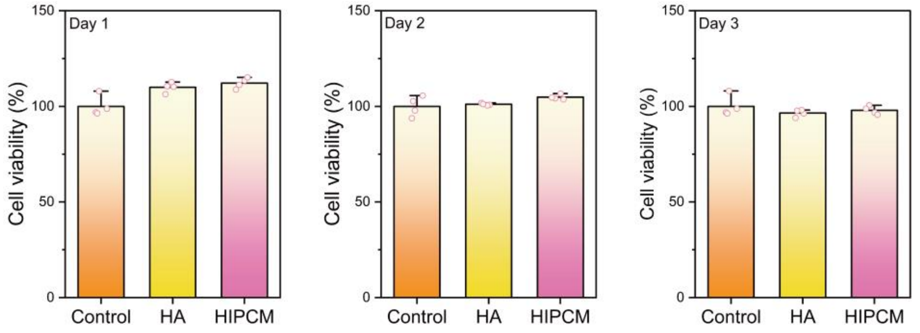

Supplementary Fig. 3. Cell viability at day 1, day 2, and day 3 (n = 4 independent replicates, data are presented as mean values ± SD). Source data are provided as a Source Data file.

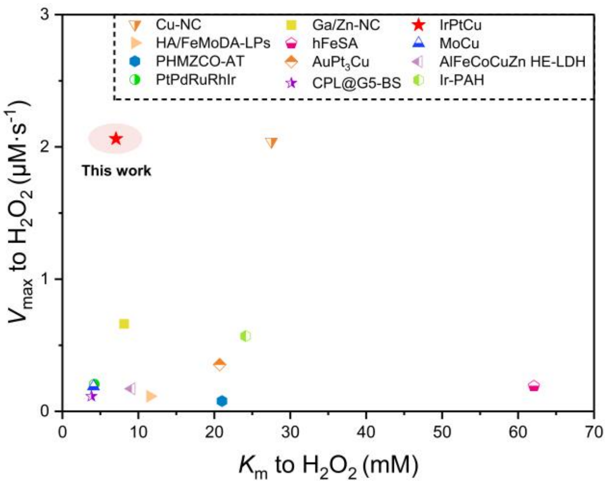

Supplementary Fig. 4. Comparison of *Km* and *Vmax* for IrPtCu nanozyme to H_2O_2 with previously reported simulated POD-like nanozymes.

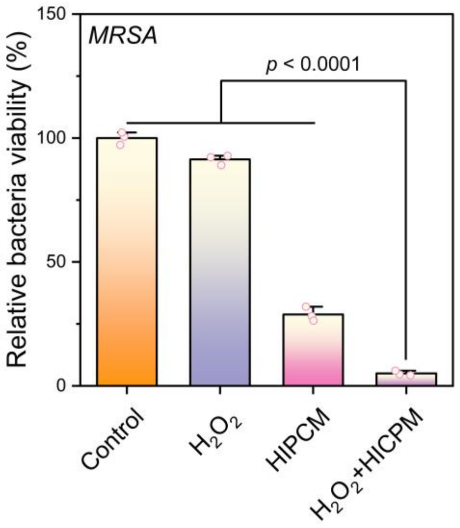

Supplementary Fig. 5. Relative bacterial viability of *MRSA* after different treatments (n = 3 independent replicates, data are presented as mean values ± SD). Statistical significance was determined using one-way ANOVA for multiple-group comparisons, followed by Tukey's two-tailed post-hoc test for pairwise analysis. Source data are provided as a Source Data file.

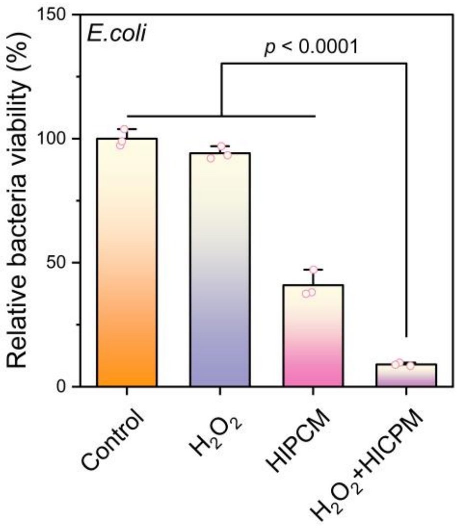

Supplementary Fig. 6. Relative bacterial viability of *E. coli* after different treatments (n = 3 independent replicates, data are presented as mean values ± SD). Statistical significance was determined using one-way ANOVA for multiple-group comparisons, followed by Tukey's two-tailed post-hoc test for pairwise analysis. Source data are provided as a Source Data file.

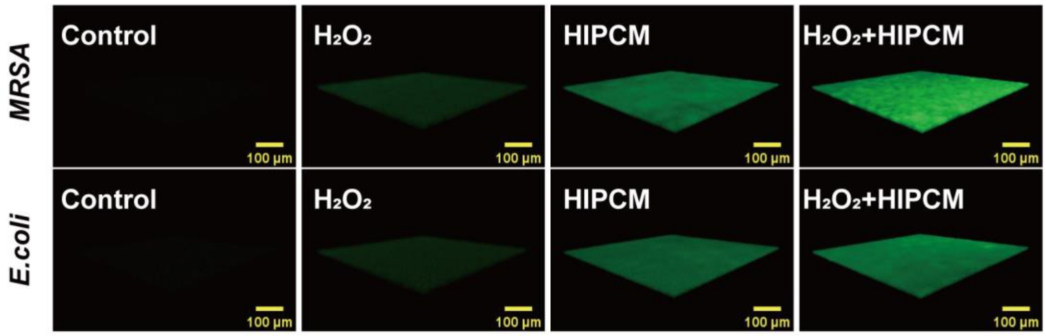

Supplementary Fig. 7. CLSM images of biofilms stained with the ROS probe DCFH-DA (Scale bars, 100 μm).

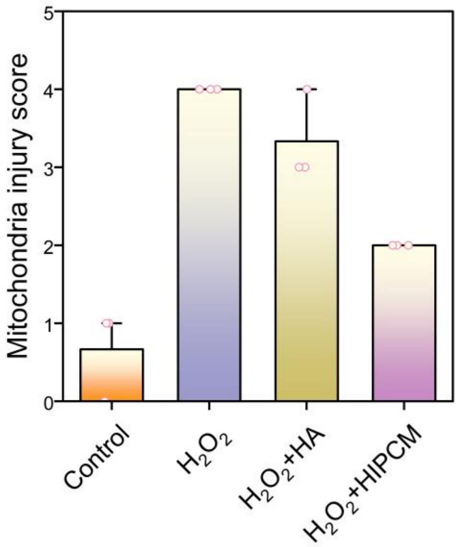

Supplementary Fig. 8. Mitochondrial injury scoring in HUVECs (n = 3 independent replicates, data are presented as mean values ± SD). Mitochondrial morphological integrity was evaluated using the Flameng method, in which ultrastructural damage was graded based on the degree of mitochondrial swelling, cristae disruption, and membrane integrity observed under transmission electron microscopy. Source data are provided as a Source Data file.

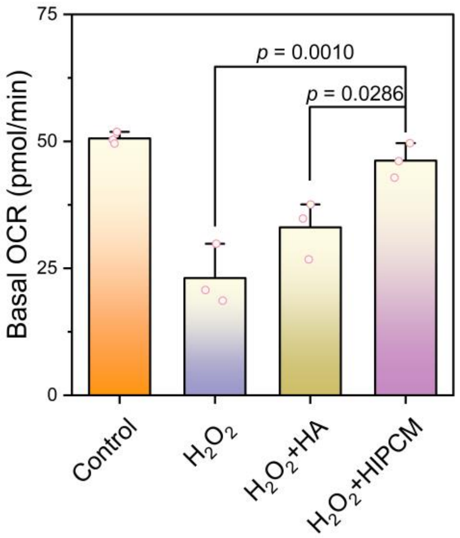

Supplementary Fig. 9. Quantitative analysis of mitochondrial basal respiration rate (n = 3 independent replicates, data are presented as mean values ± SD). Statistical significance was determined using one-way ANOVA for multiple-group comparisons, followed by Tukey's two-tailed post-hoc test for pairwise analysis. Source data are provided as a Source Data file.

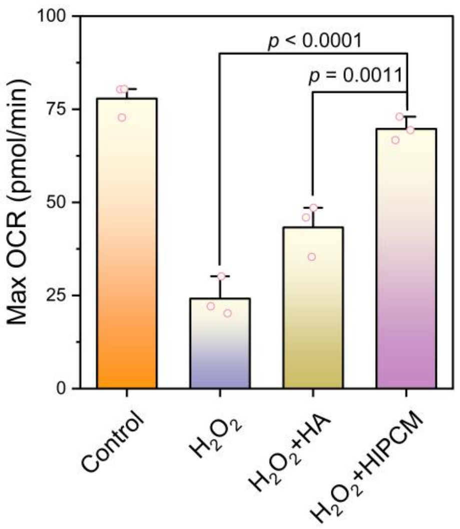

Supplementary Fig. 10. Quantitative analysis of mitochondrial ATP production rate (n = 3 independent replicates, data are presented as mean values ± SD). Statistical significance was determined using one-way ANOVA for multiple-group comparisons, followed by Tukey's two-tailed post-hoc test for pairwise analysis. Source data are provided as a Source Data file.

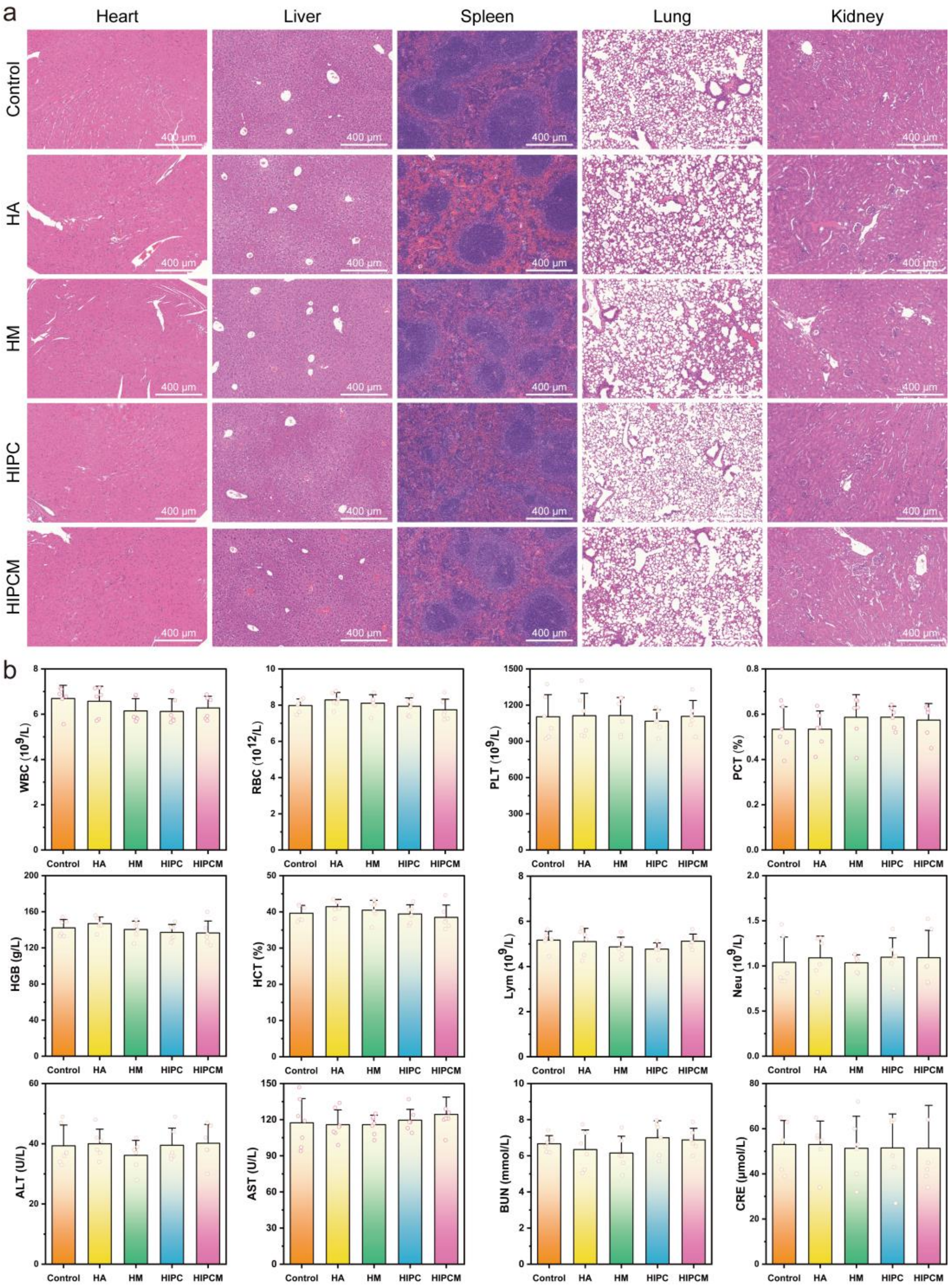

Supplementary Fig. 11. *In vivo* biocompatibility assessment of HIPCM. (a) H&E sections of major organs (the heart, liver, spleen, lungs, and kidneys) of mice in different groups on day 12 (n = 3 biologically independent mice per group; Scale bars, 400 μm). (b) Blood cell and plasma biochemistry analysis on day 12 (n = 6 biologically independent mice per group). Source data are provided as a Source Data

file.

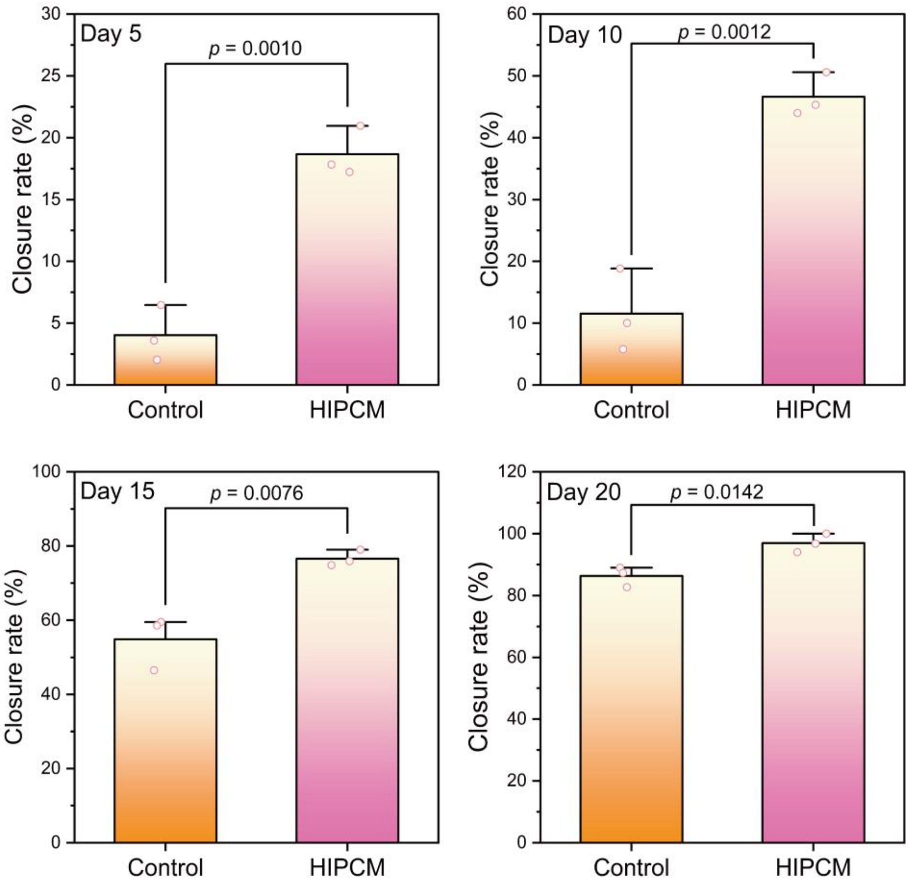

Supplementary Fig. 12. Wound healing rates under different treatment at day 5, day 10, day 15, and day 20 (n = 3 independent replicates, data are presented as mean values ± SD). All data are expressed as the mean values ± SD. Statistical significance was determined using the two-tailed Student's t-test for two-group comparisons. Source data are provided as a Source Data file.

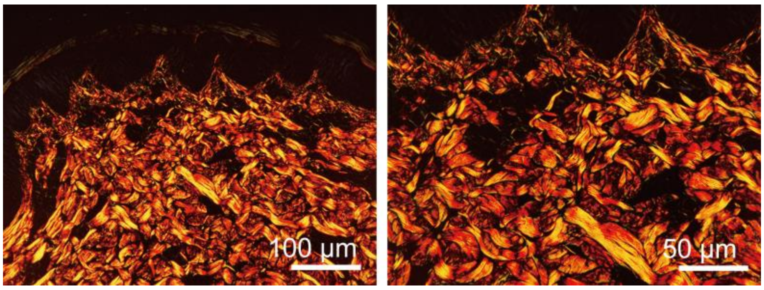

Supplementary Fig. 13. Sirius red staining in the center of the healthy skin tissue (green fluorescence indicates type III collagen, and red fluorescence represents type I

collagen, scale bars, 100 μm and 50 μm).
