# Supplementary material for: Self-adaptive nanozymes with enhanced multi-enzyme activities for sequential multimodal therapy of drug-resistant bacteria-infected wounds
Source: Nat Commun. 2026 May 28;17:6935. doi: 10.1038/s41467-026-73672-2 (PMC13389173; doi:10.1038/s41467-026-73672-2)
Supplement: Supplementary file 2 — reporting summary [file 41467_2026_73672_MOESM2_ESM.pdf]

nature portfolio

Corresponding author(s): Gan Chen, Hong Zhou, Kelong Fan, Quan Wang

Last updated by author(s): Apr 28, 2026

Reporting Summary

Nature Portfolio wishes to improve the reproducibility of the work that we publish. This form provides structure for consistency and transparency in reporting. For further information on Nature Portfolio policies, see our Editorial Policies and the Editorial Policy Checklist.

Statistics

For all statistical analyses, confirm that the following items are present in the figure legend, table legend, main text, or Methods section.

n/a	Confirmed	
☐	☒	The exact sample size (*n*) for each experimental group/condition, given as a discrete number and unit of measurement
☐	☒	A statement on whether measurements were taken from distinct samples or whether the same sample was measured repeatedly
☐	☒	The statistical test(s) used AND whether they are one- or two-sided<br>*Only common tests should be described solely by name; describe more complex techniques in the Methods section.*
☒	☐	A description of all covariates tested
☒	☐	A description of any assumptions or corrections, such as tests of normality and adjustment for multiple comparisons
☐	☒	A full description of the statistical parameters including central tendency (e.g. means) or other basic estimates (e.g. regression coefficient) AND variation (e.g. standard deviation) or associated estimates of uncertainty (e.g. confidence intervals)
☐	☒	For null hypothesis testing, the test statistic (e.g. *F*, *t*, *r*) with confidence intervals, effect sizes, degrees of freedom and *P* value noted<br>*Give P values as exact values whenever suitable.*
☒	☐	For Bayesian analysis, information on the choice of priors and Markov chain Monte Carlo settings
☒	☐	For hierarchical and complex designs, identification of the appropriate level for tests and full reporting of outcomes
☒	☐	Estimates of effect sizes (e.g. Cohen's *d*, Pearson's *r*), indicating how they were calculated

Our web collection on statistics for biologists contains articles on many of the points above.

Software and code

Policy information about availability of computer code

Data collection	The morphology and structure of IrPtCu nanozymes were characterized using a field-emission high-resolution transmission electron microscope (JEOL, JEM-F200, Japan). The morphology and structure of HIPCM were examined using a field-emission scanning electron microscope (Zeiss, Sigma 360, Germany). The elemental distribution in the IrPtCu nanozyme and HIPCM was characterized using energy-dispersive spectroscopy (EDS) mapping analysis. The elemental valence and chemical composition of the IrPtCu nanozyme were characterized using X-ray powder diffraction (Rigaku, SmartLab SE, Japan) and an X-ray photoelectron spectrometer (Thermo Scientific, K-Alpha, USA). The chemical structures were determined by Fourier transform infrared spectroscopy (Thermo Scientific, Nicolet iS20, USA). The rheological properties of the hydrogel were evaluated using a rotational rheometer (Kinexus Prime lab+, NETZSCH, Germany). Absorbance measurements were conducted with a microplate reader (SpectraMax iD5, Molecular Devices, USA). DFT calculations were performed using the Vienna Ab-initio Simulation Package (VASP). Bacterial morphology after different treatments was observed using SEM (Zeiss, Sigma 360, Germany). Blood samples were obtained for plasma biochemical analysis (MNCHIP, Celercare V5, China) and complete blood count testing (Mindray Animal Care, BC-5000 Vet, China). Confocal laser scanning microscopy (Leica STELLARIS 5, Germany) was used to acquire immunofluorescence images.
Data analysis	OriginPro 2024b, FlowJo 10.8.1, ImageJ 1.53

For manuscripts utilizing custom algorithms or software that are central to the research but not yet described in published literature, software must be made available to editors and reviewers. We strongly encourage code deposition in a community repository (e.g. GitHub). See the Nature Portfolio guidelines for submitting code & software for further information.

Data

Policy information about availability of data

All manuscripts must include a data availability statement. This statement should provide the following information, where applicable:

- Accession codes, unique identifiers, or web links for publicly available datasets
- A description of any restrictions on data availability
- For clinical datasets or third party data, please ensure that the statement adheres to our policy

All raw data generated for the figures in this study are provided in the source data file. The raw sequencing data generated in this study have been deposited in the Genome Sequence Archive (GSA; Genomics, Proteomics & Bioinformatics, 2025) in National Genomics Data Center (NGDC; Nucleic Acids Research, 2025), China National Center for Bioinformation, Chinese Academy of Sciences, under accession numbers CRA038796 (https://ngdc.cncb.ac.cn/gsa/browse/CRA038796) and CRA038863 (https://ngdc.cncb.ac.cn/gsa/browse/CRA038863).

Research involving human participants, their data, or biological material

Policy information about studies with human participants or human data. See also policy information about sex, gender (identity/presentation), and sexual orientation and race, ethnicity and racism.

Reporting on sex and gender	n/a
Reporting on race, ethnicity, or other socially relevant groupings	n/a
Population characteristics	n/a
Recruitment	n/a
Ethics oversight	n/a

Note that full information on the approval of the study protocol must also be provided in the manuscript.

Field-specific reporting

Please select the one below that is the best fit for your research. If you are not sure, read the appropriate sections before making your selection.

[x] Life sciences [ ] Behavioural & social sciences [ ] Ecological, evolutionary & environmental sciences

For a reference copy of the document with all sections, see nature.com/documents/nr-reporting-summary-flat.pdf

Life sciences study design

All studies must disclose on these points even when the disclosure is negative.

Sample size	We calculated the sample size by power analysis and determined that we required at least 3 replicates to derive an appropriate statistical test.
Data exclusions	No data were excluded from the analyses.
Replication	We confirmed that the attempts at replication were successful.
Randomization	All samples/organisms were randomly allocated into experimental groups.
Blinding	All the investigators were blinded to group allocation during data collection and analysis.

Reporting for specific materials, systems and methods

We require information from authors about some types of materials, experimental systems and methods used in many studies. Here, indicate whether each material, system or method listed is relevant to your study. If you are not sure if a list item applies to your research, read the appropriate section before selecting a response.

Materials & experimental systems

n/a	Involved in the study
[ ]	[x] Antibodies
[ ]	[x] Eukaryotic cell lines
[x]	[ ] Palaeontology and archaeology
[ ]	[x] Animals and other organisms
[x]	[ ] Clinical data
[x]	[ ] Dual use research of concern
[x]	[ ] Plants

Methods

n/a	Involved in the study
[x]	[ ] ChIP-seq
[ ]	[x] Flow cytometry
[x]	[ ] MRI-based neuroimaging

Antibodies

Antibodies used	Interleukin 6 ELISA kit (Elabscience, E-EL-M0044), interleukin 1-beta ELISA kit (Elabscience, E-EL-M0037), tumor necrosis factor alpha ELISA kit (Elabscience, E-EL-M3063),and monocyte chemotactic protein 1 ELISA Kit (Elabscience, ab204524) were used in this study. 96-well plates pre-coated with these antibodies were provided by the vendor.<br>Antibodies used for Immunofluorescence staining:<br>Anti-CD86 (Rabbit mAb, Catalog NO. GB150054-100, 1: 1000 dilution), anti-CD206 (Rabbit mAb, Catalog NO. GB153497-100, 1: 1000 dilution), anti-alpha smooth muscle (Rabbit pAb, Catalog NO. GB111364-100, 1: 500 dilution), anti-CD31 (Mouse mAb, Catalog NO. GB15063-100, 1: 500 dilution), anti-HIF-1 alpha (Rabbit mAb, Catalog NO. GB151339-100, 1: 1000 dilution), anti-Ki67 (Mouse mAb, Catalog NO. GB121141-100, 1: 500 dilution), and anti-CK19 (Rabbit mAb, Catalog NO. GB15197-100, 1: 500 dilution) were purchased by Wuhan Servicebio Technology Co., Ltd.(China).
Validation	anti-CD86: https://www.servicebio.cn/goodsdetail?id=24231<br>anti-CD206: https://www.servicebio.cn/goodsdetail?id=24947<br>anti-alpha smooth muscle: https://www.servicebio.cn/goodsdetail?id=3743<br>anti-CD31: https://www.servicebio.cn/goodsdetail?id=21920<br>anti-HIF-1 alpha: https://www.servicebio.cn/goodsdetail?id=23626<br>anti-Ki67: https://www.servicebio.cn/goodsdetail?id=6801<br>anti-CK19: https://www.servicebio.cn/goodsdetail?id=24431<br>All antibodies were verified by the supplier and each lot has been quality tested.

Eukaryotic cell lines

Policy information about cell lines and Sex and Gender in Research

Cell line source(s)	L929 (mouse fibroblast), HUVECs (human umbilical vein endothelial cells), and RAW 264.7 (mouse macrophage) cell lines were obtained from the American Type Culture Collection (ATCC; Manassas, VA, USA).
Authentication	A short tandem repeat DNA profiling method was used to authenticate the cell lines and the results were compared with reference database.
Mycoplasma contamination	No mycoplasma contamination was found.
Commonly misidentified lines (See ICLAC register)	No commonly misidentified cell lines were used in this study.

Animals and other research organisms

Policy information about studies involving animals; ARRIVE guidelines recommended for reporting animal research, and Sex and Gender in Research

Laboratory animals	ICR mice (male, 8 weeks, 25-28 g) were purchased from Charles River Laboratories Co., Ltd. Bama mini pigs (male, 10kg) were purchased from Baoding Longxiang Laboratory Animal Breeding Co., Ltd. The mice and pigs are housed under a 12-hour light/12-hour dark cycle, with the environmental temperature maintained at 22°C ± 2°C and the humidity generally controlled between 40% and 60%.
Wild animals	No wild animals were used in this study.
Reporting on sex	Male mice were used in this study.
Field-collected samples	No field collected samples were used in the study.
Ethics oversight	All the animal experiments were conducted in compliance with the Chinese National Standard and under the protocols that were approved by the Animal Ethics Committee of the Academy of Military Medical Sciences (IACUC – DWZX – 2024 – P536).

Note that full information on the approval of the study protocol must also be provided in the manuscript.

Plants

Seed stocks	n/a
Novel plant genotypes	n/a
Authentication	n/a

Flow Cytometry

Plots

Confirm that:

- [x] The axis labels state the marker and fluorochrome used (e.g. CD4-FITC).
- [x] The axis scales are clearly visible. Include numbers along axes only for bottom left plot of group (a 'group' is an analysis of identical markers).
- [x] All plots are contour plots with outliers or pseudocolor plots.
- [x] A numerical value for number of cells or percentage (with statistics) is provided.

Methodology

Sample preparation	For the LPS-stimulated RAW 264.7 macrophage model, cells were seeded in 6-well plates and cultured for 24 h, followed by incubation with complete DMEM containing LPS (500 ng /mL) and hydrogel extracts (500 µg/ mL) for another 24 h. For the H2O2-stimulated HUVECs model, cells were seeded in 6-well plates, cultured for 24 h, and then treated with complete DMEM containing H2O2 (200 µM) and hydrogel extracts (500 µg /mL) for 4 h to induce oxidative stress. After treatment, cells were gently washed with PBS and incubated with DCFH-DA probe according to the manufacturer's general guidelines (10 µM, 30 min, 37 °C, in the dark). The labeled cells were then rinsed to remove excess dye, harvested, and immediately analyzed by flow cytometry to quantify intracellular ROS levels.
Instrument	Flow Cytometric Cell Sorter (Becton, Dickinson and Company, BD FACSAria™ II, USA)
Software	FlowJo 10.8.1
Cell population abundance	10,000 cells were counted and analyzed in all experiments.
Gating strategy	Flow cytometric data were analyzed using FlowJo 10.8.1. On a FSC-A vs SSC-A scatter plot, events representing small particles and debris (characterized by both low FSC-A and low SSC-A) were excluded. The main population corresponding to RAW264.7 or HUVEC cells was identified based on their characteristic size (FSC) and internal complexity (SSC) and gated for subsequent analysis.

- [x] Tick this box to confirm that a figure exemplifying the gating strategy is provided in the Supplementary Information.
