# Supplementary material for: Self-adaptive nanozymes with enhanced multi-enzyme activities for sequential multimodal therapy of drug-resistant bacteria-infected wounds
Source: Nat Commun. 2026 May 28;17:6935. doi: 10.1038/s41467-026-73672-2 (PMC13389173; doi:10.1038/s41467-026-73672-2)
Supplement: Supplementary file 3 — Transparent Peer Review file [file 41467_2026_73672_MOESM3_ESM.pdf]

nature portfolio

Peer Review File

Self-Adaptive Nanozymes with Enhanced Multi-Enzyme Activities for Sequential Multimodal Therapy of Drug-Resistant Bacteria-Infected Wounds

Corresponding Author: Professor Gan Chen

This file contains all reviewer reports in order by version, followed by all author rebuttals in order by version.

Version 0:

Reviewer comments:

Reviewer #1

(Remarks to the Author)
This is an interesting manuscript by Zhang et al. that constructs an effective and comprehensive strategy integrating self-adaptive engineered nanozymes with plant-derived natural herbal medicines, providing valuable insights for the development of future therapies against drug-resistant bacteria-infected wounds. The authors present a comprehensive set of experiments, including material synthesis and physicochemical characterization, catalytic kinetics and mechanistic studies, as well as therapeutic evaluation in both small and large animal models. Overall, the manuscript is well prepared and would likely be of considerable interest to the readership of Nature Communications. Here are some minor issues:

In the SAED patterns, the scale bar is labeled in nm-1. Please clarify and standardize the unit notation to avoid ambiguity. In addition, ensure consistency in scale bar color across all figures.

The formatting of P values is inconsistent throughout the manuscript, particularly in the transcriptomic analyses. Please standardize the format and verify that the statistical annotations (e.g., asterisks) in the text correspond precisely to those shown in the figures.

In Fig. 4, the quantitative analysis of live/dead bacterial staining lacks clarity. The authors should explicitly define which experimental groups correspond to labels 1-4.

In Figs. 2 and 3, K_m and Vmax should be presented in italic typeface, in accordance with standard biochemical notation. In Fig. 9, some axis titles show inconsistent or redundant capitalization and should be carefully revised.

The abstract is overly long and would benefit from substantial condensation to improve readabilit.

The IrPtCu nanozyme exhibits multiple enzyme mimicking activities. The authors are encouraged to more explicitly link these activities to the observed biological outcomes in the Results and Discussion sections, thereby strengthening the mechanistic interpretation.

The criteria used for mitochondrial damage scoring are not described. The authors should provide a clear definition of the scoring system or cite an appropriate reference.

In the minipig full thickness skin defect model, it is unclear whether wound closure rates were quantitatively analyzed and whether statistically significant differences were observed among groups. Please provide quantitative data and statistical analysis, if available.

For the blood cell counts and plasma biochemistry analyses, the authors should clarify whether any significant differences were detected between groups and explicitly annotate or discuss these results.

Reviewer #2

(Remarks to the Author)
The treatment of infected wounds constitutes a major clinical challenge, and to address this unmet need, Zhang et al. developed a HA-based smart hydrogel system that co-delivers IrPtCu nanozymes with dual ROS-regulating properties and madecassoside, a bioactive component derived from traditional Chinese medicine. This system is designed for the treatment of MRSA-infected wounds and full-thickness skin defects. Utilizing a self-adaptive microenvironment strategy that integrates anti-infection, anti-inflammation, and tissue regeneration, the study demonstrates effective infection control, resolution of inflammation, and reconstruction of tissue in both murine and miniature pig models, indicating considerable translational potential. Overall, the study is well-conceived and experimentally solid. However, several issues should be addressed to further improve the clarity, rigor, and impact of the manuscript.

1. For the miniature pig experiments, it is recommended to include Sirius Red-stained polarized light images of normal skin as a reference to visually illustrate the ordered remodeling of collagen fibers. In addition, please emphasize in the discussion section the translational advantages and clinical relevance of the miniature pig model.

2. The integration of computational modeling with experimental validation represents a major strength of this work. However, the correlation between the computational results and biological experiments is currently underexplained. It would be helpful to provide a concise clarification of this relationship, especially for readers with limited computational background, to enhance understanding of how both approaches mutually support the study's conclusions.

3. In the discussion, it is advisable to highlight the unique combination of stage-adaptive healing and plant-derived active compound. Elaborate on the specific mechanistic role of madecassoside, such as its contribution to ordered collagen deposition and angiogenesis. Emphasizing its synergistic action during the repair process would further underscore the innovation and significance of this study.

4. Please clearly state the sample size (n) for all quantitative experiments either in the main text or figure captions, and specify the statistical methods used, including any corrections for multiple comparisons. These details are essential for enhancing the statistical reliability and transparency of the results.

5. The representation of macrophage inflammatory factor changes in Scheme 1 is not sufficiently intuitive. Consider using clearer symbols or quantitative indicators to improve readability. Additionally, ensure that the text colors used to explain mechanisms in different healing phases are more distinguishable to facilitate visual interpretation.

6. While the overall writing is clear, the manuscript would benefit from further refinement to improve logical flow and readability. Several sentences are overly long and information-dense. Consider breaking down lengthy sentences to avoid redundancy and adjective accumulation. For instance, in the abstract, the phrase "This study presents an effective comprehensive strategy…" could be streamlined to strengthen clarity and readability, allowing readers to grasp the core contribution more efficiently.

7. Please include the manufacturer and country information for all key instruments to enhance reproducibility and transparency. In the figures, unify the scale bar color scheme and improve font legibility. Particular attention should be paid to Figure 7i, ensuring consistent positioning and appearance of the scale bar.

Reviewer #3

(Remarks to the Author)
This study develops novel anti-infection hydrogel based on IrPtCu nanozyme and natural herbal medicine. IrPtCu nanozyme shows unique pH-adaptive ROS regulation properties, which realizes ROS-generation in the infection stage and ROS-elimination in the healing state. The in vivo experimental results by using MRSA-infected mouse and pig wound models highlights the superior therapeutic efficacy between the nanozyme and madecassoside, providing a promising strategy for treating drug-resistant bacterial infections. This is quite a unique system with potential to address difficult-to-treat chronic infections as demonstrated with both mouse and pig infection models. The manuscript itself is well-organized and written. Therefore, the manuscript is recommended for publication following minor revisions.
Questions and concerns need to be addressed:
1. In Scheme 1, the wound location in the mini-pig model does not match that shown in the subsequent schematic diagram.
2. The choice of atomic ratio of Ir:Pt:Cu should be given more detailed discussion.
3. IrPtCu nanozymes exhibit six types of enzyme-mimicking activities, it is recommended to further clarify the chain linking structure, function, and biological effects.
4. The nanozyme shows multi-enzyme-like activities. Does their any relation-ships between the contents and the specific enzyme activity? Further discussion should be supplemented.
5. In Figure 4a, only representative colony images are provided. Please include quantitative antibacterial rate measurements to support the findings.
6. In Figure 7, the image resolution should be improved for better interpretation, and the legend in Figure 7a contains a description error that needs correction.
7. Please specify the statistical methods in detail, including the tests used for multiple-group comparisons and the post hoc test applied.

8. The abbreviation for madecassoside in both the main text and figure legends should be checked for consistency and appropriateness.
9. For the mini-pig experiment, please clarify whether there is a statistically significant difference in wound healing rate between the HIPCM and control groups, and provide corresponding statistical analysis if applicable.
10. In Figure 9, “relative college content” should be corrected to “relative collagen content”. Additionally, ensure abbreviation capitalization is consistent across the figure.

Version 1:

Reviewer comments:

Reviewer #1

(Remarks to the Author)
The author has completed the revisions as requested and has met the standards of the journal.

Reviewer #2

(Remarks to the Author)
The authors have carefully revised the manuscript, all my concerns have been replied. It can be accepted now.

Reviewer #3

(Remarks to the Author)
The authors have addressed all the questions and carefully revised the manuscript. I have no more qustions about the manuscript.

Open Access This Peer Review File is licensed under a Creative Commons Attribution 4.0 International License, which permits use, sharing, adaptation, distribution and reproduction in any medium or format, as long as you give appropriate credit to the original author(s) and the source, provide a link to the Creative Commons license, and indicate if changes were made.
In cases where reviewers are anonymous, credit should be given to 'Anonymous Referee' and the source.
The images or other third party material in this Peer Review File are included in the article’s Creative Commons license, unless indicated otherwise in a credit line to the material. If material is not included in the article’s Creative Commons license and your intended use is not permitted by statutory regulation or exceeds the permitted use, you will need to obtain permission directly from the copyright holder.
To view a copy of this license, visit https://creativecommons.org/licenses/by/4.0/

Point-by-point responses the review comments

Reviewer comments:

Reviewer #1:

This is an interesting manuscript by Zhang et al. that constructs an effective and comprehensive strategy integrating self-adaptive engineered nanozymes with plant-derived natural herbal medicines, providing valuable insights for the development of future therapies against drug-resistant bacteria-infected wounds. The authors present a comprehensive set of experiments, including material synthesis and physicochemical characterization, catalytic kinetics and mechanistic studies, as well as therapeutic evaluation in both small and large animal models. Overall, the manuscript is well prepared and would likely be of considerable interest to the readership of Nature Communications. Here are some minor issues:

Response: We sincerely appreciate the reviewer's positive comments and professional suggestions regarding our work. We have carefully revised the manuscript in accordance with these recommendations, and our detailed responses to each comment are presented below.

1. In the SAED patterns, the scale bar is labeled in nm^{-1}. Please clarify and standardize the unit notation to avoid ambiguity. In addition, ensure consistency in scale bar color across all figures.

Response: We sincerely thank the reviewer for this valuable suggestion. We have carefully revised the label in SAED patterns to ensure that the unit notation is clearly presented as nm^{-1} throughout the manuscript. Furthermore, the scale bar color has been standardized across all figures for visual consistency and clarity. These modifications have been incorporated in the revised version (see Fig. 2 on page 10).

2. The formatting of P values is inconsistent throughout the manuscript, particularly in the transcriptomic analyses. Please standardize the format and verify that the

statistical annotations (e.g., asterisks) in the text correspond precisely to those shown in the figures.

Response: We thank the reviewer for pointing out this important issue. In the revised manuscript, all p values have been standardized to a consistent format throughout the text, tables, and figures, following conventional statistical reporting guidelines. In addition, all statistical annotations, including asterisks indicating significance levels, have been carefully checked to ensure that they exactly correspond between the text descriptions and the figures.

3. In Fig. 4, the quantitative analysis of live/dead bacterial staining lacks clarity. The authors should explicitly define which experimental groups correspond to labels 1-4.

Response: We sincerely appreciate the reviewer's helpful comment. We have revised the caption of Figure 4 and the corresponding description in the main text to clearly define the experimental groups labeled as 1-4. Specifically, labels 1-4 now correspond to 1: Control, 2: H_2O_2, 3: HIPCM, and 4: H_2O_2+HIPCM, respectively.

4. In Figs. 2 and 3, K_m and Vmax should be presented in italic typeface, in accordance with standard biochemical notation. In Fig. 9, some axis titles show inconsistent or redundant capitalization and should be carefully revised.

Response: In the revised manuscript, the symbols K_m and V_{max} in Figure 2 and 3 have been reformatted in italics to comply with standard biochemical notation. Additionally, all axis titles in Figure 9 have been carefully checked and corrected to ensure consistent capitalization and proper formatting.

5. The abstract is overly long and would benefit from substantial condensation to improve readability.

Response: We appreciate the reviewer's valuable suggestion. In the revised manuscript, the abstract has been simplified and condensed to improve clarity and readability.

6. The IrPtCu nanozyme exhibits multiple enzyme mimicking activities. The authors are encouraged to more explicitly link these activities to the observed biological outcomes in the Results and Discussion sections, thereby strengthening the mechanistic interpretation.

Response: We appreciate the suggestion. The Discussion section has been revised to more clearly correlate each enzyme-mimicking activity with the corresponding antibacterial and wound-healing outcomes (see paragraph 1 on page 36).

7. The criteria used for mitochondrial damage scoring are not described. The authors should provide a clear definition of the scoring system or cite an appropriate reference.

Response: We thank the reviewer for this helpful suggestion. In the revised manuscript, we have now included a detailed description of the mitochondrial damage scoring system in the Mitochondrial Morphological Analysis section (see paragraph 4 on page 44).

page 45: *Mitochondrial morphological integrity was assessed following the Flameng method. Briefly, ultrathin sections of tissue samples were prepared and examined using TEM. Mitochondrial ultrastructural alterations were evaluated and graded according to the extent of mitochondrial swelling, disruption of cristae, and compromise of membrane integrity, providing a quantitative measure of mitochondrial damage.*

8. In the minipig full thickness skin defect model, it is unclear whether wound closure rates were quantitatively analyzed and whether statistically significant differences were observed among groups. Please provide quantitative data and statistical analysis, if available.

Response: We appreciate the reviewer’s insightful comment. Quantitative analysis of wound closure rates was performed at days 5, 10, 15, and 20 in the minipig full-thickness skin defect model. Significant differences were observed among groups at days 5, 10, and 15 ($p < 0.01$) and at day 20 ($p < 0.05$). We have now clarified this

information in the Results section and referenced Supplementary Fig. 12 for detailed quantitative data.

Updated Supplementary Fig. 12:

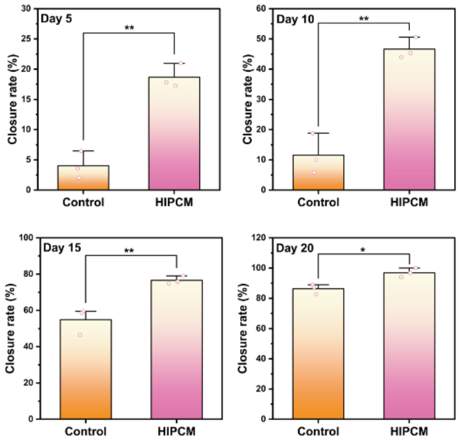

Supplementary Fig. 12 Wound healing rates under different treatment at day 5, day 10, day 15, and day 20 (n = 3 independent replicates). All data are expressed as the mean values ± SD. Statistical significance was determined using the Student's t-test for two-group comparisons, p value: *$p < 0.05$, **$p < 0.01$, ***$p < 0.001$.

9. For the blood cell counts and plasma biochemistry analyses, the authors should clarify whether any significant differences were detected between groups and explicitly annotate or discuss these results.

Response: We thank the reviewer for this helpful suggestion. No significant differences in blood cell counts or plasma biochemistry parameters were detected among the groups. We have added this information to the Results section on page 27, second paragraph, specifying that all values remained within the normal physiological range without statistically significant variation between groups.

page 27： *To evaluate their biocompatibility of HIPCM for in vivo therapeutic applications, histological analysis of the heart, liver, spleen, lung, and kidney was performed after 12 days of treatment, with no significant tissue abnormalities observed (Supplementary Fig. 11). In addition, there were no significant differences in physiological blood parameters among the groups, indicating favorable in vivo biocompatibility of HIPCM (Supplementary Fig. 11). Collectively, these results*

demonstrate that HIPCM can serve as a high-performance antibacterial wound treatment material.

Reviewer #2 (Remarks to the Author):

The treatment of infected wounds constitutes a major clinical challenge, and to address this unmet need, Zhang et al. developed a HA-based smart hydrogel system that co-delivers IrPtCu nanozymes with dual ROS-regulating properties and madecassoside, a bioactive component derived from traditional Chinese medicine. This system is designed for the treatment of MRSA-infected wounds and full-thickness skin defects. Utilizing a self-adaptive microenvironment strategy that integrates anti-infection, anti-inflammation, and tissue regeneration, the study demonstrates effective infection control, resolution of inflammation, and reconstruction of tissue in both murine and miniature pig models, indicating considerable translational potential. Overall, the study is well-conceived and experimentally solid. However, several issues should be addressed to further improve the clarity, rigor, and impact of the manuscript.

Response: We are truly grateful to the reviewer for the valuable and positive feedback on our work We have carefully revised the manuscript in accordance with these recommendations, and our detailed responses to each comment are presented below.

1. For the miniature pig experiments, it is recommended to include Sirius Red-stained polarized light images of normal skin as a reference to visually illustrate the ordered remodeling of collagen fibers. In addition, please emphasize in the discussion section the translational advantages and clinical relevance of the miniature pig model.

Response: We thank you for this valuable suggestion. Sirius Red-stained polarized light images of normal skin have been added as a reference in Supplementary Fig. 13 to illustrate the organized arrangement of collagen fibers. In addition, the Discussion section has been revised to further highlight the translational advantages of the pig

model (see paragraph 1 on page 31 and paragraph 1 on page 38).

Updated Supplementary Fig. 13:

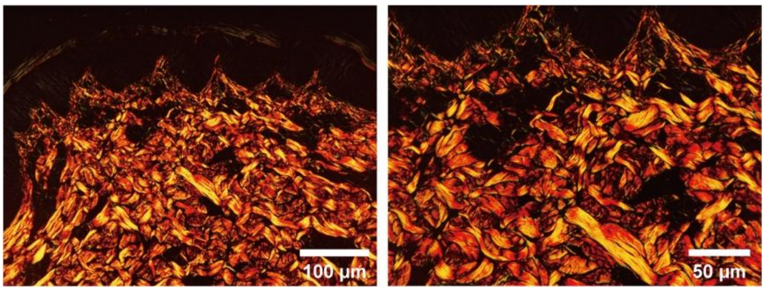

Supplementary Fig. 13. Sirius red staining in the center of the healthy skin tissue (green fluorescence indicates type III collagen, and red fluorescence represents type I collagen, scale bars, 100 μm and 50 μm).

2. The integration of computational modeling with experimental validation represents a major strength of this work. However, the correlation between the computational results and biological experiments is currently underexplained. It would be helpful to provide a concise clarification of this relationship, especially for readers with limited computational background, to enhance understanding of how both approaches mutually support the study's conclusions.

Response: We appreciate the reviewer's insightful comment. A new paragraph has been added to explicitly clarify the relationship between the DFT calculations and the experimental observations. Specifically, the Ir incorporation-induced electronic modulation revealed by the DFT results accounts for the experimentally observed enhancement in ROS generation and antibacterial performance (see paragraph 2 on page 35).

3. In the discussion, it is advisable to highlight the unique combination of stage-adaptive healing and plant-derived active compound. Elaborate on the specific mechanistic role of madecassoside, such as its contribution to ordered collagen deposition and angiogenesis. Emphasizing its synergistic action during the repair process would further underscore the innovation and significance of this study.

Response: As recommended, we strengthened the discussion section to emphasize the synergistic action between the pH-responsive IrPtCu nanozyme and madecassoside. Madecassoside facilitates ordered collagen deposition and angiogenesis, cooperating with nanozyme-mediated oxidative stress regulation to achieve stage-adaptive tissue repair (see paragraph 2 on page 37).

4. Please clearly state the sample size (n) for all quantitative experiments either in the main text or figure captions, and specify the statistical methods used, including any corrections for multiple comparisons. These details are essential for enhancing the statistical reliability and transparency of the results.

Response: We appreciate the reviewer's constructive suggestion. The sample size (n) for all quantitative experiments and the statistical methods, including corrections for multiple comparisons when applicable, have been clearly indicated in the figure captions and described in detail in the Statistical Analysis section on page 47.

page 47： *Data analysis was conducted using OriginPro 2024b, ImageJ 1.53 was utilized for the quantitative analysis of in vitro and in vivo imaging. Quantitative data were collected at least three independent times. All data are expressed as the mean values ± SD. Statistical significance was determined using the Student's t-test for two-group comparisons, and one-way ANOVA for multiple-group comparisons, followed by Tukey's two-tailed post-hoc test for pairwise analysis, all tests were two-sided. p value: *p < 0.05, **p < 0.01, ***p < 0.001.*

5. The representation of macrophage inflammatory factor changes in Scheme 1 is not sufficiently intuitive. Consider using clearer symbols or quantitative indicators to improve readability. Additionally, ensure that the text colors used to explain mechanisms in different healing phases are more distinguishable to facilitate visual interpretation.

Response: We thank the reviewer for the helpful suggestion. Scheme 1 has been revised to present macrophage inflammatory factor changes using clearer quantitative indicators and more intuitive graphical symbols, thereby improving overall readability.

In addition, the text colors corresponding to different healing phases have been adjusted to enhance visual distinction and facilitate interpretation.

Updated Fig. 1:

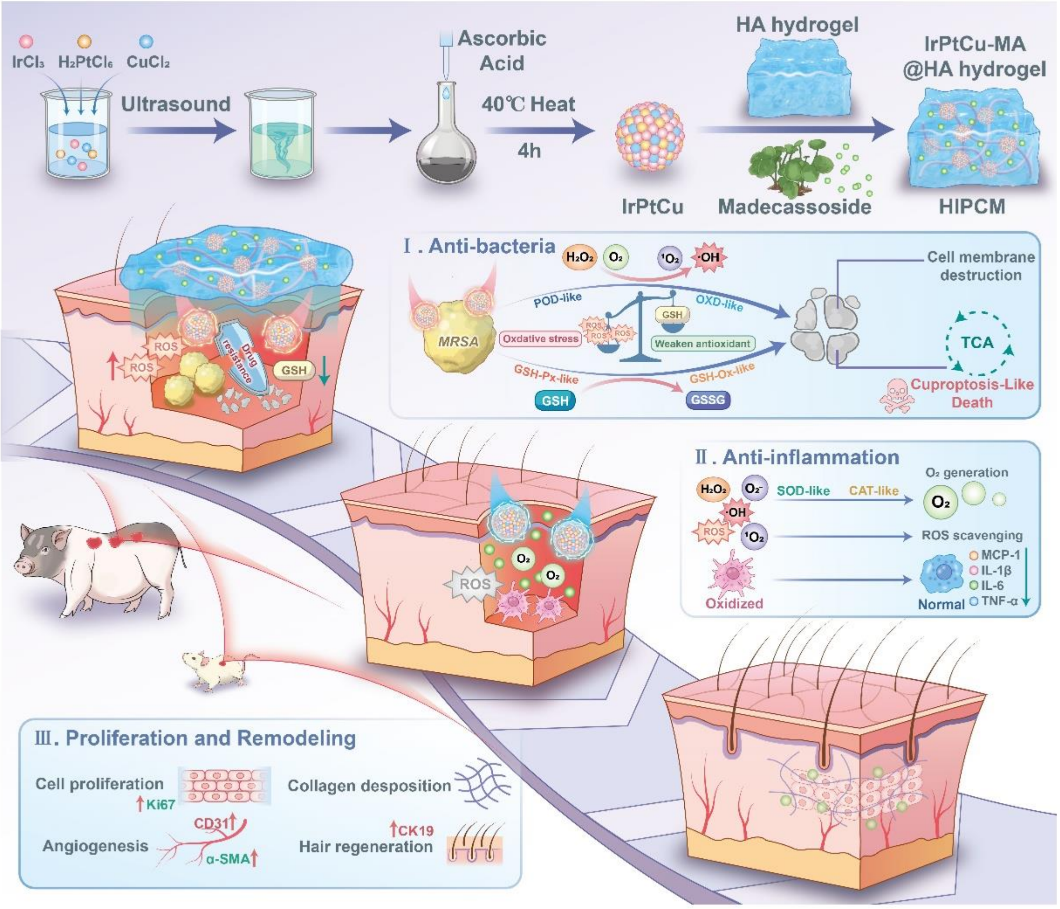

6. While the overall writing is clear, the manuscript would benefit from further refinement to improve logical flow and readability. Several sentences are overly long and information-dense. Consider breaking down lengthy sentences to avoid redundancy and adjective accumulation. For instance, in the abstract, the phrase “This study presents an effective comprehensive strategy…” could be streamlined to strengthen clarity and readability, allowing readers to grasp the core contribution more efficiently.

Response: We greatly appreciate the reviewer’s valuable comments on improving the manuscript’s logical flow and readability. We carefully reviewed the entire text and

revised lengthy or information-dense sentences to achieve a more concise and coherent presentation.

7. Please include the manufacturer and country information for all key instruments to enhance reproducibility and transparency. In the figures, unify the scale bar color scheme and improve font legibility. Particular attention should be paid to Figure 7i, ensuring consistent positioning and appearance of the scale bar.

Response: We thank the reviewer for the constructive suggestions. The manuscript has been revised to include detailed manufacturer names and country information for all key instruments to improve reproducibility and transparency. In addition, the scale bar color scheme has been unified across all figures, and the font style and size have been adjusted to enhance legibility. Figure 7i has been specifically refined to ensure consistent scale bar positioning and appearance with the other figures.

Reviewer #3 (Remarks to the Author):

This study develops novel anti-infection hydrogel based on IrPtCu nanozyme and natural herbal medicine. IrPtCu nanozyme shows unique pH-adaptive ROS regulation properties, which realizes ROS-generation in the infection stage and ROS-elimination in the healing state. The in vivo experimental results by using MRSA-infected mouse and pig wound models highlights the superior therapeutic efficacy between the nanozyme and madecassoside, providing a promising strategy for treating drug-resistant bacterial infections. This is quite a unique system with potential to address difficult-to-treat chronic infections as demonstrated with both mouse and pig infection models. The manuscript itself is well-organized and written. Therefore, the manuscript is recommended for publication following minor revisions.

Response: We really appreciate the very positive remarks and valuable suggestions from the reviewer. We have carefully revised the manuscript in accordance with these recommendations, and our detailed responses to each comment are presented below.

Questions and concerns need to be addressed:

1. In Scheme 1, the wound location in the mini-pig model does not match that shown in the subsequent schematic diagram.

Response: We appreciate the reviewer's careful observation. The inconsistency in the wound location between Scheme 1 and the subsequent schematic diagram has been corrected. The revised Scheme 1 now accurately reflects the wound position in accordance with the experimental setup and the schematic description, ensuring visual consistency and accuracy throughout the manuscript.

Updated Fig. 1:

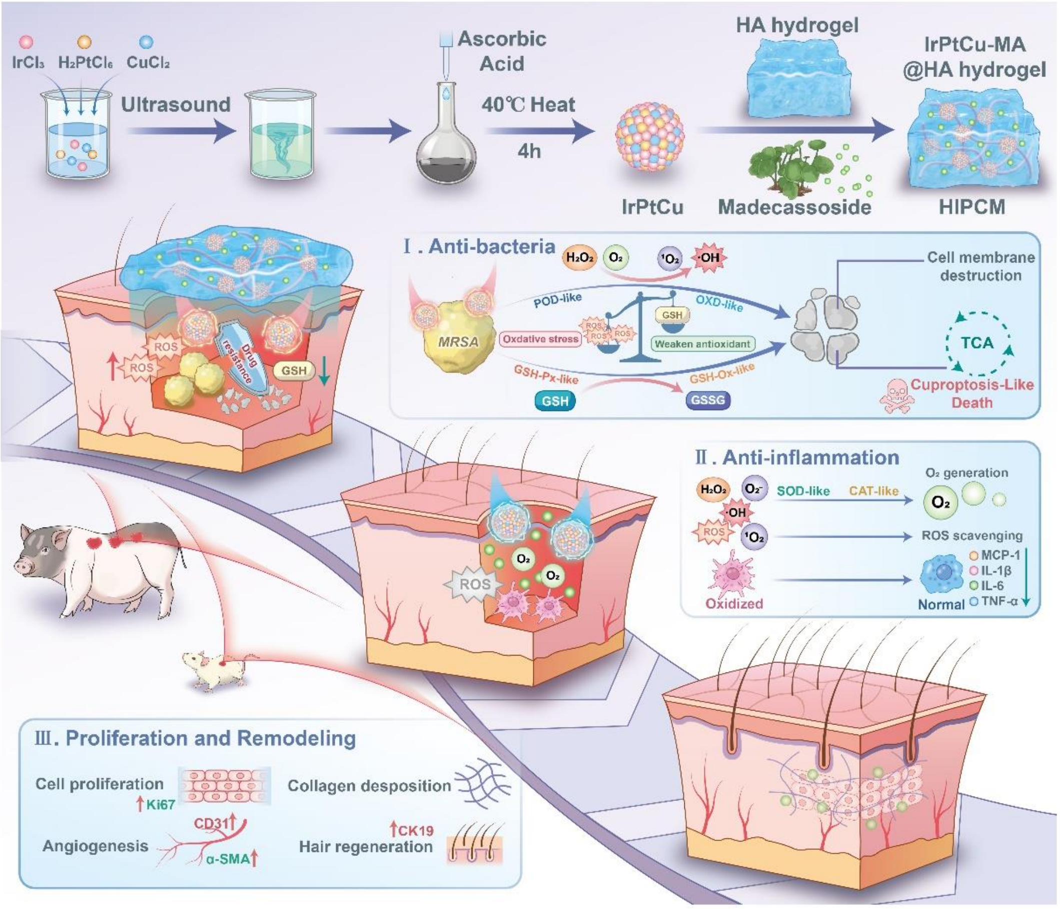

2. The choice of atomic ratio of Ir:Pt:Cu should be given more detailed discussion.

Response: We appreciate the reviewer's constructive suggestion. The ratio of Pt to Cu was chosen based on previously reported literature (Yu-Qing Liu, et al. Nano Today

36, 101027, 2021). On this basis, different $IrCl_3$ addition concentrations (0-20 mM) were further introduced during synthesis to investigate their effects on the POD- and CAT-like activities. After systematic testing, the optimal Ir precursor concentration was determined to be 10 mM, which provided the highest catalytic activity. The corresponding results have been added in Supplementary Fig. 1.

Updated Supplementary Fig. 1:

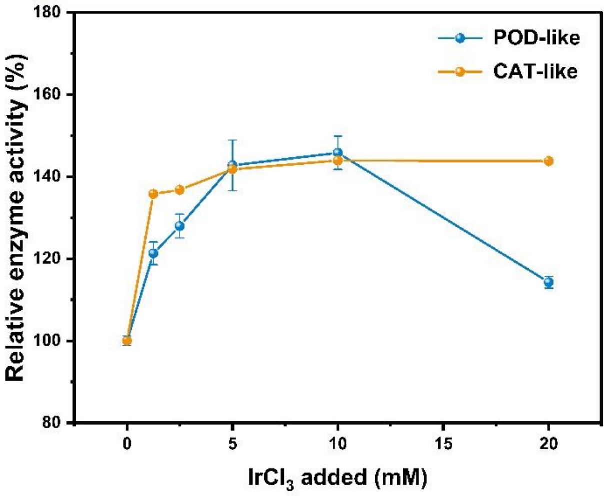

Supplementary Fig. 1. Effect of different $IrCl_3$ addition concentrations on the POD- and CAT-like activities of IrPtCu nanozyme.

3. IrPtCu nanozymes exhibit six types of enzyme-mimicking activities, it is recommended to further clarify the chain linking structure, function, and biological effects.

Response: We have revised the Discussion section to explicitly illustrate how structural design (Ir-modulated electronic configuration) determines catalytic function and results in sequential biological responses, forming a coherent structure-function-effect cascade (see paragraph 1 on page 36).

4. The nanozyme shows multi-enzyme-like activities. Does their any relation-ships between the contents and the specific enzyme activity? Further discussion should be supplemented.

Response: We appreciate the reviewer's insightful comment. Pt provides catalytic sites for ROS reactions, Cu contributes to antibacterial interactions, and Ir facilitates

the adsorption and activation of H_2O_2 and simultaneously catalyzes the efficient oxidation of GSH to GSSG. Their balanced ratio collectively modulates multi-enzyme activities performance. This explanation has been added to the revised manuscript (see paragraph 1 on page 36).

5. In Figure 4a, only representative colony images are provided. Please include quantitative antibacterial rate measurements to support the findings.

Response: We thank the reviewer for this valuable suggestion. Quantitative antibacterial-rate data have now been provided. The detailed statistical analysis of antibacterial efficiency is presented in Supplementary Fig. 5 and 6. These results further confirm the antibacterial performance demonstrated in Fig. 5.

Supplementary Fig. 5 and 6:

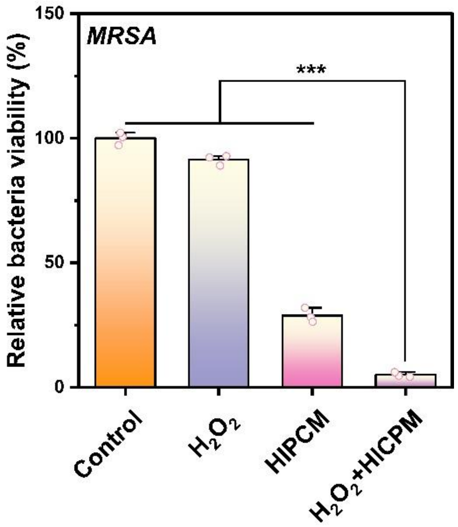

Supplementary Fig. 5. Relative bacterial viability of *MRSA* after different treatments (n = 3 independent replicates). Statistical significance was determined using one-way ANOVA for multiple-group comparisons, followed by Tukey's two-tailed post-hoc test for pairwise analysis, all tests were two-sided. p value: ***$p < 0.001$.

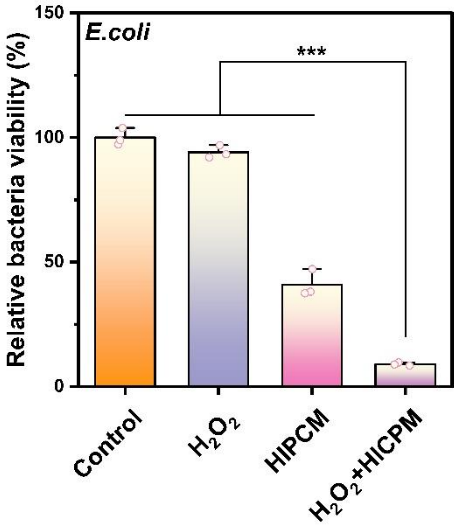

Supplementary Fig. 6. Relative bacterial viability of *E. coli* after different treatments (n = 3 independent replicates). Statistical significance was determined using one-way ANOVA for multiple-group comparisons, followed by Tukey's two-tailed post-hoc test for pairwise analysis, all tests were two-sided. p value: ***$p < 0.001$.

6. In Figure 7, the image resolution should be improved for better interpretation, and the legend in Figure 7a contains a description error that needs correction.

Response: We appreciate the reviewer's helpful comment. The resolution of all images in Figure 7 has been enhanced to ensure clear visualization and accurate interpretation of the results. In addition, the description error in the legend of Figure 7a has been carefully revised to correctly represent the corresponding experimental details. The updated figure and legend are now consistent and have been included in the revised manuscript.

7. Please specify the statistical methods in detail, including the tests used for multiple-group comparisons and the post hoc test applied.

Response: We thank the reviewer for this important suggestion. The statistical analysis section has been revised to provide detailed information. In addition, corresponding details have been supplemented in the figure legends. The modifications to the corresponding section have been highlighted in red.

page 47： *Data analysis was conducted using OriginPro 2024b, ImageJ 1.53 was utilized for the quantitative analysis of in vitro and in vivo imaging. Quantitative data were collected at least three independent times. All data are expressed as the mean values ± SD. Statistical significance was determined using the Student's t-test for two-group comparisons, and one-way ANOVA for multiple-group comparisons, followed by Tukey's two-tailed post-hoc test for pairwise analysis, all tests were two-sided. p value: *$p < 0.05$, **$p < 0.01$, ***$p < 0.001$.*

8. The abbreviation for madecassoside in both the main text and figure legends should be checked for consistency and appropriateness.

Response: We appreciate the reviewer's valuable suggestion. The abbreviation for madecassoside has been reviewed throughout the manuscript, including the main text and all figure legends. To ensure consistency and clarity, we have uniformly changed the abbreviation to MA.

9. For the mini-pig experiment, please clarify whether there is a statistically significant difference in wound healing rate between the HIPCM and control groups, and provide corresponding statistical analysis if applicable.

Response: We thank the reviewer for this helpful comment. The wound healing rates of the HIPCM and control groups were statistically compared. Significant differences were observed among groups at days 5, 10, and 15 ($p < 0.01$) and at day 20 ($p < 0.05$). We have now clarified this information in the Results section and referenced Supplementary Fig. 12 for detailed quantitative data.

Updated Supplementary Fig. 12:

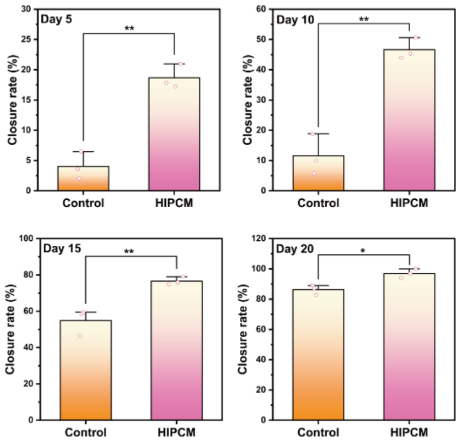

Supplementary Fig. 12. Wound healing rates under different treatment at day 5, day 10, day 15, and day 20 (n = 3 independent replicates). All data are expressed as the mean values ± SD. Statistical significance was determined using the Student's t-test for two-group comparisons, p value: *$p < 0.05$, **$p < 0.01$, ***$p < 0.001$.

10. In Figure 9, "relative college content" should be corrected to "relative collagen content". Additionally, ensure abbreviation capitalization is consistent across the figure.

Response: We appreciate the reviewer's careful observation. The description error in Figure 9 has been corrected from "relative college content" to "relative collagen content." In addition, all abbreviations in the figure and its legend have been checked and revised to ensure consistent capitalization and formatting throughout.
